# The Baker's Yeast Diploid Genome Is Remarkably Stable in Vegetative Growth and Meiosis

**DOI:** 10.1371/journal.pgen.1001109

**Published:** 2010-09-09

**Authors:** K. T. Nishant, Wu Wei, Eugenio Mancera, Juan Lucas Argueso, Andreas Schlattl, Nicolas Delhomme, Xin Ma, Carlos D. Bustamante, Jan O. Korbel, Zhenglong Gu, Lars M. Steinmetz, Eric Alani

**Affiliations:** 1 Department of Molecular Biology and Genetics, Cornell University, Ithaca, New York, United States of America; 2 European Molecular Biology Laboratory, Heidelberg, Germany; 3 Department of Molecular Genetics and Microbiology, Duke University Medical Center, Durham, North Carolina, United States of America; 4 Department of Biological Statistics and Computational Biology, Cornell University, Ithaca, New York, United States of America; 5 Department of Genetics, Stanford University, Stanford, California, United States of America; 6 Division of Nutritional Sciences, Cornell University, Ithaca, New York, United States of America; Duke University, United States of America

## Abstract

Accurate estimates of mutation rates provide critical information to analyze genome evolution and organism fitness. We used whole-genome DNA sequencing, pulse-field gel electrophoresis, and comparative genome hybridization to determine mutation rates in diploid vegetative and meiotic mutation accumulation lines of *Saccharomyces cerevisiae*. The vegetative lines underwent only mitotic divisions while the meiotic lines underwent a meiotic cycle every ∼20 vegetative divisions. Similar base substitution rates were estimated for both lines. Given our experimental design, these measures indicated that the meiotic mutation rate is within the range of being equal to zero to being 55-fold higher than the vegetative rate. Mutations detected in vegetative lines were all heterozygous while those in meiotic lines were homozygous. A quantitative analysis of intra-tetrad mating events in the meiotic lines showed that inter-spore mating is primarily responsible for rapidly fixing mutations to homozygosity as well as for removing mutations. We did not observe 1–2 nt insertion/deletion (in-del) mutations in any of the sequenced lines and only one structural variant in a non-telomeric location was found. However, a large number of structural variations in subtelomeric sequences were seen in both vegetative and meiotic lines that did not affect viability. Our results indicate that the diploid yeast nuclear genome is remarkably stable during the vegetative and meiotic cell cycles and support the hypothesis that peripheral regions of chromosomes are more dynamic than gene-rich central sections where structural rearrangements could be deleterious. This work also provides an improved estimate for the mutational load carried by diploid organisms.

## Author Summary

Mutations result from errors that occur during DNA metabolism. They provide the raw materials for evolution, can affect organism fitness, and have been shown to accumulate in organisms during asexual growth. During a sexual life cycle, mutations can be removed by recombination and mating. While such removal is thought to provide a fitness advantage, studies have shown that recombination itself is mutagenic. To examine if the mutation rate in an organism differs during asexual and sexual cycles, we sequenced the entire nuclear genome of lines of diploid baker's yeast that underwent only asexual growth, or alternating cycles of asexual and sexual growth. The estimated rate of base substitutions in the vegetative lines was extremely low (2.9×10^−10^ base substitutions per base per cell generation) and the meiotic mutation rate is within the range of being equal to zero to being 55 times higher than the vegetative rate. Interestingly, we observed a large number of changes in the ends of chromosomes in the asexual and sexual cycles that did not affect fitness; changes at other locations were very rare, suggesting a remarkable genome stability of diploid baker's yeast.

## Introduction

Mutations can arise in genomes as the result of errors that occur during DNA replication, and the repair of DNA lesions [1], [2]. Mutations such as base substitutions, small insertions and deletions, and large-scale rearrangements are raw materials for adaptive evolution [3]–[5]; however, the deleterious nature of most mutations imposes a fitness cost. In asexual organisms deleterious mutations can accumulate in successive generations. This phenomenon, known as Muller's ratchet, can cause a continuous decrease in fitness and population size in small asexual populations [6]–[8]. In sexual organisms, deleterious mutations can be removed from the population by meiotic recombination and mating [6], [9]. While this removal of mutations is thought to provide a fitness advantage for sexual organisms, several studies have shown that recombination is itself mutagenic [10]–[12]. Meiosis can also generate new allelic combinations [13], thus increasing genetic variation and the rate of adaptation to new environments [14]. Therefore, obtaining accurate estimates of mutation rate in vegetative and meiotic cell cycles is important for understanding disease progression, genome evolution, species divergence times and patterns of selection (reviewed in [15], [16]). These measures also improve our estimates of the mutational load carried by organisms, which are crucial to understand the evolutionary role of sex and recombination.

Several genome-wide measurements have been performed to determine the vegetative base substitution rate in a variety of organisms (reviewed in [16]). In baker's yeast, for example, the base substitution rate in haploid mutation accumulation lines grown vegetatively was estimated to be 3.3×10^−10^ substitutions per base per cell division [17]. Importantly, there are no genome wide estimates of the meiotic mutation rates in any organism. However, several lines of correlative and experimental evidence suggest that mutation rates in meiosis are higher than in vegetative growth. First, the phenotypic reversion rates of three independent mutations in *S. cerevisiae* were observed to be six to twenty-fold higher in meiosis compared to vegetative growth [10]. Second, several studies showed a high mutation rate (≥100-fold elevated) associated with DSB repair of a broken chromosome [11], [12], [18], [19]. Mutations are thought to occur due to error-prone DNA synthesis and/or the absence, or lack of bias, of DNA mismatch repair. Although the mutation rate estimates are for vegetative DSB repair, homologous recombination in meiosis is initiated by the programmed introduction of DSBs [20]–[21]. Lastly, a positive correlation between genetic diversity and meiotic recombination rates has been observed in several organisms [22]–[27]. Curiously, Noor [28] did not see an association between recombination hotspots or DSB sites and sequence divergence between two yeast species (lack of a correlation, or a negative correlation). A concern about most correlation analyses is that they assume that DSB sites are conserved between individuals of the same species and among species. For yeasts, conservation of meiotic DSB sites was recently reported between different species [29].

In this study we used deep DNA sequencing, pulse-field gel electrophoresis (PFGE), and comparative genome hybridization (CGH) to determine nuclear mutation rates in vegetative growth and meiosis in diploid mutation accumulation lines of *S. cerevisiae*. *S. cerevisiae* is an ideal model organism to obtain such rates because it undergoes rapid vegetative growth (∼2 hr cell cycle) and can complete meiosis in ∼10 hours. Wild isolates of *S. cerevisiae* are mostly diploid [30], [31]; importantly, diploid strains can maintain recessive lethal mutations that can comprise 30% to 40% of deleterious mutations [32], [33]. Vegetative lines were subjected to bottlenecks, from one cell to a colony, every 20 generations, for a total of ∼1740 generations. The meiotic lines underwent 50 meioses and 1,000 intervening vegetative generations. While this scheme made it difficult to directly estimate meiotic mutation rates, it was compatible with work indicating that the meiotic cycle is infrequent (for *Saccharomyces paradoxus* one meiotic cycle/1,000 vegetative cycles; [34]). Such a scheme also provides information on how mutations created in the vegetative cycle are propagated as the result of meiosis. As described below, our observations indicate that the baker's yeast diploid genome is highly stable in the vegetative and meiotic cell cycles.

## Results

### Experimental approach

To measure vegetative and meiotic mutation rates in the nuclear genome, we performed mutation accumulation studies in the SK1 homothallic strain of yeast, which grows rapidly in rich media and can complete meiosis in approximately 10 hours [35]. The starting strain for this work, EAY2531 (relevant genotype *MATa/MATalpha*, *HO/HO*), is, with exception of the *MAT* locus, fully homozygous. The spore viabilities of tetrads derived from EAY2531 are greater than 95%. EAY2531 was sequenced using both single and paired end approaches covering 96% of the genome at 64-fold average coverage (Materials and Methods; Table S1). Data can be retrieved from the European Nucleotide Archive (http://www.ebi.ac.uk/ena) using the accession number: ERA007227. The high sequence coverage allowed us to assemble a high quality reference SK1 genome, accessed in http://steinmetzlab.embl.de/SK1.

**Figure 1:**
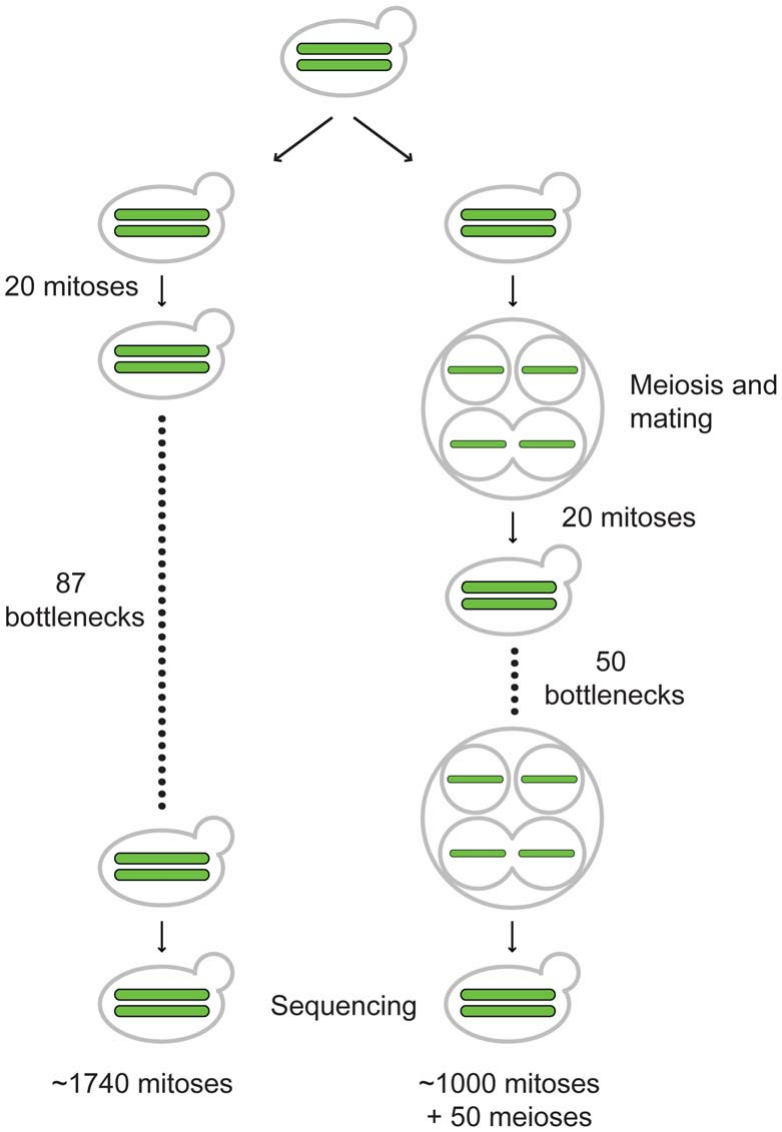
Outline of vegetative and meiotic bottlenecks. EAY2531 (relevant genotype *MATa/MATalpha*, *HO/HO*) was struck to single cells and then grown for 20 generations on YPD media to form single colonies. 20 such independent colonies were split into pairs of vegetative and meiotic mutation accumulation lines (one representative line shown for each). For the vegetative lines, a colony for each line was struck to single cells. This process was repeated 87 times to achieve ∼1740 generations of growth. At the end of generation 1740, a colony for each of the 20 independent lines was sporulated, and four haploid spores derived from each line were germinated and grown on YPD media to isolate chromosomal DNA for whole-genome sequencing. The 20 starting independent colonies of EAY2531 described above were also sporulated. One tetrad from each line was isolated and then germinated on YPD media and grown for 20 generations to form a colony. Each colony contained almost exclusively diploid cells as the result of intra-spore (shown here) and self-mating. For each line, the colony was then sporulated and the bottleneck was repeated 50 times. This yielded lines that were maintained for ∼1,000 vegetative generations, with one round of meiosis every 20 vegetative generations.

Vegetative and meiotic mutation accumulation lines were initiated from EAY2531 (Materials and Methods; Figure 1). Twenty vegetative lines labeled 1B to 20B were subjected to vegetative growth bottlenecks, from one cell to a colony, every 20 generations for a total of ∼1740 generations (87 bottlenecks). The twenty meiotic lines labeled 1T to 20T were subject to a bottleneck every meiosis by isolating one complete tetrad that was separately germinated to form colonies. The resulting colony was sporulated and the bottleneck was repeated for 50 meioses and 1,000 intervening vegetative generations. At the end of the bottleneck experiments, cells from the final B (1B-87 to 20B-87) and T (1T-50 to 20T-50) generations were sporulated and tetrad dissected to assess fitness. As shown in Table S2, all of the meiotic lines displayed spore viabilities similar to the parental line, indicating that they had not acquired recessive lethal mutations or they had been removed by recombination and mating. Furthermore, we examined ten of the meiotic lines at intermediate stages of the meiotic bottleneck (T-10, 20, 30, 40). All of the lines displayed spore viability similar to the parental line. In contrast, three of twenty vegetative lines displayed spore viabilities consistent with the accumulation of a single recessive lethal mutation. Such a result is consistent with vegetative lines accumulating heterozygous mutations (see below). Vegetative and meiotic lines were examined for the presence of mutations using deep sequencing, PFGE, and CGH.

### Determination of base substitution mutation rates in vegetative (B) and meiotic (T) lines using whole-genome DNA sequencing analysis

To provide an estimate of vegetative and meiotic mutation rates in diploid yeast, whole genome paired end sequencing was performed for the mitotic 3B-87 and 4B-87 lines, and for the meiotic 3T-50 and 4T-50 lines (Materials and Methods). For the 3B-87, 4B-87 and 4T-50 lines a single haploid spore clone was isolated from a complete tetrad from the final bottlenecks, germinated and grown in culture. For the 3T-50 line three spores from a complete tetrad were germinated and grown in culture. The parental strain, EAY2531, was sequenced as a diploid because no heterozygosities apart from the *MAT* locus were expected; none were detected by sequencing. We also sequenced the diploid genome of the 2B line at generation 52 (2B-52, ∼1040 generations) using a single end approach. The sequencing coverage is presented in Table S1. For the vegetative lines, eight, six, and five base substitutions were identified in 3B-87, 4B-87, and 2B-52, respectively (Table 1). The nineteen base substitutions were verified by Sanger sequencing of DNA isolated from 3B-87, 4B-87, and 2B-52 diploids (Materials and Methods). This analysis also confirmed that sporulating the lines at the end of the bottlenecks did not introduce new mutations. All nineteen substitutions were heterozygous in the diploid lines; this was expected because they were propagated clonally in the absence of a sexual cycle. For the 3B-87 and 4B-87 lines half of the genome was sequenced because only one spore clone was analyzed; thus to determine the genome-wide mutation rate for these two lines, we multiplied by two the number of base substitutions detected. After this correction we estimate that the single base substitution rates in the vegetative 3B87, 4B-87 and 2B-52 lines were 3.8×10^−10^, 2.8×10^−10^ and 2.0×10^−10^ substitutions per base per cell division, respectively (24,483,546 bp genome at 96% coverage for 1740 (87 bottlenecks) or 1040 (52 bottlenecks) generations). The average of these rates, 2.9×10^−10^ per base per cell division, is very similar to values obtained by Lynch *et al.* [17] in a haploid mutation accumulation study (3.3×10^−10^), and by Drake [36] who estimated base substitution mutation rates in haploid yeast at the *CAN1* (1.7×10^−10^) and *URA3* (2.8×10^−10^) loci.

**Table 1:** Genome location of derived mutations in the B87 and T50 lines.

Line	Mutation	SGD position	Gene, amino acid change
2B-52	A>G	ChrIII, 145135	*CWH43*, L832L
	G>A	ChrIV, 1062644	*PRO1*, H49Y
	G>C	ChrVIII, 150074	*YHR022C*, F89V
	G>T	ChrX, 631351	*CPA2*, N527K
	C>T	ChrX, 673974	*SGM1*, L623L
3B-87	G>T	ChrI, 177541	
	A>C	ChrIII, 117835	*CDC10*, I171R
	G>T	ChrIV, 962798	*PAM1*, A730S
	C>A	ChrIV, 1026680	*GCN2*, D1123Y
	T>A	ChrV, 563878	
	G>A	ChrXIII, 209683	*SRC1*, Q53Q
	C>A	ChrXVI, 545073	*NCR1*, A149E
	G>A	ChrXVI, 656427	*YPR045C*, L42L
4B-87	A>G	ChrIV, 66310	
	G>A	ChrIV, 66758	*YDL218W*, A89T
	A>G	ChrX, 193151	*PHO86*, N208S
	C>A	ChrX, 471267	*BNA1*, V133F
	T>G	ChrXII, 706989	*ECI1*, P18P
	G>T	ChrXIII, 670507	*INP1*, S273Y
3T-50	A>G	ChrIV, 180916	*MSH5*, K861R
	G>T	ChrIV, 601543	*SED1*, L91F
	A>G	ChrIX, 290215	
	G>A	ChrX, 677807	*TTI2*, T107I
	A>T	ChrXVI, 187332	*NAB3*, E131D, null is inviable
4T-50	G>T	ChrIII, 156792	*HSP30*, S104S
	C>T	ChrXII, 793815	
	C>A	ChrXIII, 534158	*RRB1*, L180F, null inviable
	G>T	ChrXV, 45147	*DCP1*, D71Y, null inviable
	C>A	ChrXV, 226642	

Single base mutations identified in haploid spores from the vegetative (3B-87, 4B-87) and meiotic (3T-50, 4T-50) lines and in diploids from the vegetative (2B-52) line. All mutations in the vegetative lines were heterozygous and all mutations in the meiotic lines were homozygous. Single base mutations observed in the B and T lines were annotated relative to the S288C reference genome (*Saccharomyces* genome data base (SGD); http://www.yeastgenome.org). The SGD coordinate and the amino acid change due to the mutation are shown. Deletion phenotype of the gene, if inviable, is also indicated.

For the meiotic line 3T-50 the same five base substitutions were detected in genomic DNA isolated from each of the three sequenced spore clones. This information, in conjunction with Sanger sequencing from 3T-50 diploid cells, indicated that the five base substitutions were homozygous in the final bottleneck. The one spore sequenced from the 4T-50 line also contained five base substitutions (Table 1). Sanger sequencing from 4T-50 diploid cells indicated that these five base substitutions were also homozygous in the final bottleneck. Because all mutations were homozygous in the meiotic lines, we did not need to correct for the total number of base substitutions, even for the 4T-50 line where we only sequenced one spore. However, to determine the base substitution rate, we multiplied the number of base substitutions in each line by two to account for the loss of half of the base substitutions accumulated in the vegetative phase of the bottleneck during intra-tetrad mating (see below). Based on this assumption, both lines showed the same base substitution rate, 3.9×10^−10^ per base per cell division (10 base substitutions per line in a 24,483,546 bp genome grown for 1,000 vegetative and 50 meiotic generations). This value is nearly identical to that obtained for the vegetative base substitution rate estimate.

Most mutations in the vegetative and meiotic lines (17/29) were in coding regions and resulted in non-synonymous substitutions (Table 1). Of the nineteen base substitution mutations detected in vegetative lines, eight were transitions and eleven were transversions (Table 1). Twelve of these mutations resulted in a change from a G-C to an A-T base pair, whereas only five were in the opposite direction. For the ten base substitutions seen in the meiotic lines, four were transitions and six were transversions (Table 1). Seven of these resulted in a change from a G-C to an A-T base pair, whereas only two were in the opposite direction. The overall bias towards A-T base pairs was seen and discussed previously (e.g. [17], [37], [38]).

### Simulations to estimate the meiotic mutation rate

**Figure 2:**
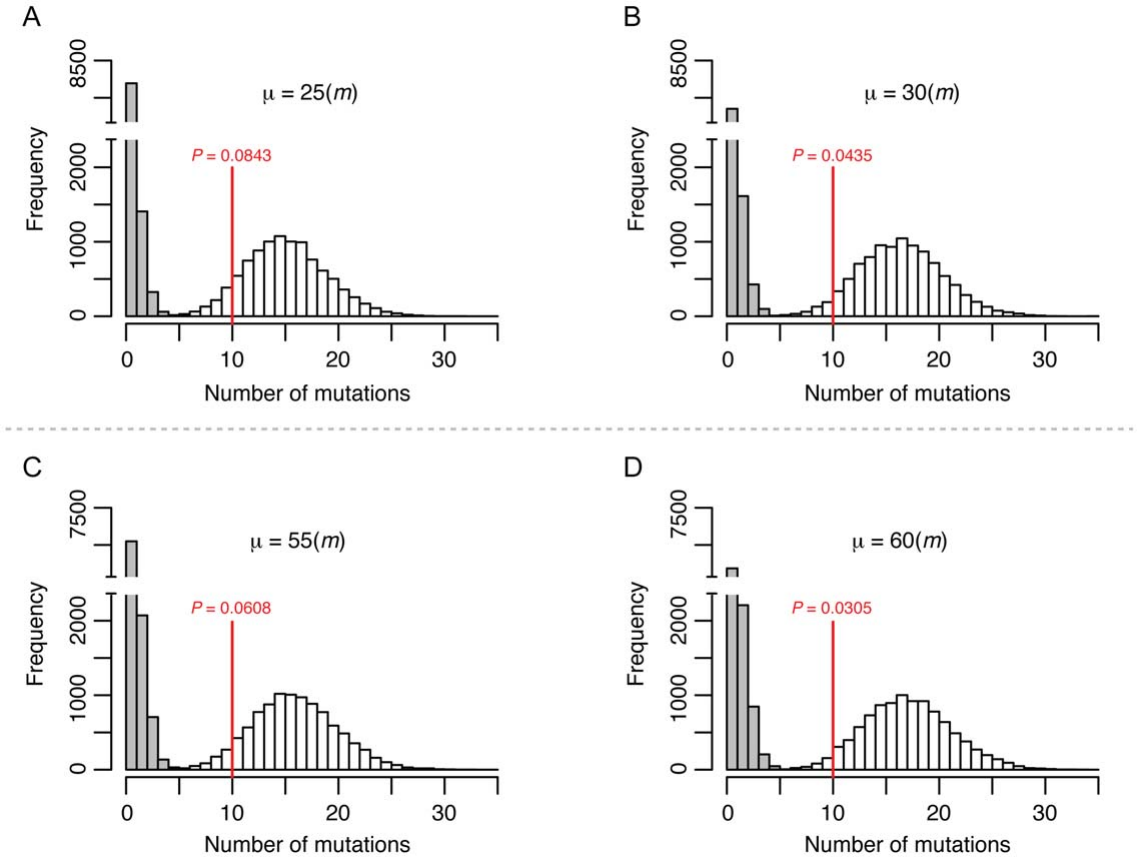
Simulation to estimate the upper limit for the meiotic mutation rate. The histograms show the distribution of the final number of homozygous (white) and heterozygous (grey) mutations occurred in 10,000 independent simulated lines after 1,000 mitotic divisions and 50 meiotic bottlenecks in each line. The putative meiotic mutation rate (*μ*) used for each of the simulation is shown relative to the mitotic mutation rate (*m*). The red vertical lines show the average number of SNPs (all homozygous) observed in the T-50 lines. The *P*-value denotes the frequency of simulations with equal or lower number of SNPs than the observed value. Panel A and B show simulations in which meiotic mutations were set to occur before DNA replication and therefore are present in two chromatids. Panels C and D show simulations in which meiotic mutations were set to occur during or after DNA replication and are therefore present in one single chromatid. See Material and Methods and Figure S1 for further details on the simulations.

The fact that we did not observe significant differences between the base substitution rates of the mitotic and meiotic lines could reflect the relatively low number of meiotic (50) compared to vegetative divisions (1,000) in the meiotic bottlenecks. To estimate the upper limit of the meiotic mutation rate we simulated the occurrence of mutations given different meiotic mutation rates and taking into account the experimental setup. The rates obtained by simulation were compared to the observed rates to establish a range of meiotic mutation rates consistent with the observed values. We considered two scenarios in this analysis (Figure S1). In the first, mutations occurred prior to meiotic DNA replication and are thus present in two of the four chromatids of a homolog. In the second, mutations occur during or after meiotic DNA replication (during double strand break repair) and are present in only one of the four chromatids. In both scenarios we accounted for the spore self-mating frequency that was experimentally determined (17%, see below). As shown in Figure 2A and 2B and Figure S2A, in the first scenario the distribution of simulated mutations became statistically different from the observed meiotic rate (*P*<0.05) when the simulated meiotic mutation rate (μ) was 30-fold higher than the vegetative rate (*m*). This shows that the meiotic mutation rate is only consistent with the observed rates if it is within the range of being equal to zero to being 30-fold higher than the vegetative rate. In the second scenario (Figure 2C and 2D, Figure S2B), the meiotic mutation rate can be around 55-fold higher than the vegetative rate and still be consistent with our observations; if it was higher than that we would have observed a difference between the rates of the two mutation accumulation schemes. Although our experiments do not allow exact determination of the meiotic mutation rate they show that this rate can be no higher than ∼1.74×10^−8^ per base per cell division in *S. cerevisiae*.

### Short in-del mutations and intermediate-sized structural variants are extremely rare in vegetative and meiotic lines

To identify 1–2 nt in-del mutations, we aligned the sequencing reads obtained for all of the sequenced lines against the reference genome SK1 using the Novoalign software (Materials and Methods; http://www.novocraft.com). Statistical methods were performed to identify high confidence 1–2 nt in-del mutations (XM and CB, unpublished; Materials and Methods). We did not detect such in-dels in any of the sequenced lines. A second approach to identify in-dels by aligning the reads to the S288c sequenced genome also did not reveal any in-dels specific to the mutation accumulation lines (see Materials and Methods).

To search for intermediate-sized structural variants (SV; >500 bp), we analyzed positional discrepancies between paired-end reads [39] and performed read depth coverage analysis [40], [41]. The SV predictions were validated using real-time quantitative PCR (qPCR), Southern blotting, and PCR (Table S3; Figure S3; Materials and Methods). In paired end mapping, SVs larger than a cutoff of approximately 500–1,000 bp (depending on the insert size distribution, see Materials and Methods) can be identified. However, pair-end mapping did not identify SVs that were specific to the sequenced mutation accumulation lines. Read depth analysis can identify SVs larger than 900 bp (see Materials and Methods). Only one of 55 potential SVs identified by read depth analysis was verified by both qPCR and Southern analysis (Figure S3; data not shown). A region (∼3.0 KB) that showed high similarity to the Ty3 element, a relatively rare class of retrotransposon present in yeast (two copies in S288c; [42]), was present at higher abundance in 3T50 than in the parental strain, suggesting the gain of at least one copy. Southern analysis showed that a new Ty3 element was inserted into the ribosomal DNA cluster on chromosome XII in the 3T-50 isolate (data not shown). The location of the retrotransposition was determined by PCR and Sanger sequencing (Figure S3). While we were successful in identifying a Ty3 retrotransposition event, it is important to note that our read depth analysis does not have the sensitivity to detect copy number variation associated with transposition of more abundant repetitive elements such as Ty1 or Ty2 (∼50 copies in S288c; [42]). It is also not possible to detect SVs of between three and 500 bp with our short-read data. However, the low number of intermediate sized SVs found is surprising given previous measures of gene duplication and gene loss in haploid mutation accumulation lines of yeast ([17]; see Discussion).

### Distinct large-scale structural variations confined to chromosome ends occur in the vegetative and meiotic lines

In addition to whole genome re-sequencing of specific mutation accumulation lines, we investigated the occurrence of gross chromosomal rearrangements in all vegetative (20) and meiotic (19) lines by using PFGE to resolve full-length chromosomes (Figure S4, Figure S5). As summarized in Table 2, the chromosomal rearrangements detected in the two strain sets were similar in both their high abundance (∼75% of lines had at least one visible size change) and their large-scale deviation from the respective parental chromosomes (±10 to 40 KB). In both sub-culturing regimens, Chromosome IX was the least stable chromosome (∼50% of all size changes), with ten cases detected in the meiotic lines and eleven in the vegetative lines. While we frequently observed heterozygous changes in the vegetative lines (i.e. two homologs of different size could be distinguished), in the meiotic lines, all but 1 of the 24 instances of the size changes were present in both homologs of the affected chromosome, presumably due to loss of heterozygosity through meiotic inbreeding (see below). We also saw an increase in chromosome size in the meiotic lines (seventeen chromosome sizes increased and seven decreased) compared to the vegetative lines (seven increased and ten decreased), but this difference was not statistically significant (*P* = 0.11, Fisher's Exact Test). Finally, we also noted that changes in the meiotic lines involved a more diverse set of chromosomes than in the vegetative lines (seven chromosomes vs. three chromosomes, respectively).

**Table 2:** Summary of chromosome size changes detected by PFGE karyotyping.

	Lines with detectable changes in chromosome size^a^	Types of size changes			Chromosomes
		increase	decrease	heterozygous^b^	
Vegetative lines	15/20	7	10	11/17	V,VIII,IX
Meiotic lines	14/19	17	7	1/24	I,II,V,VI,VIII,IX,X

a Data compiled from the combined analysis of the PFGE karyotypes in Figures 3, S4, and S5.

b Indicates the number of cases where two homologous chromosomes of different size can be distinguished in the PFGE karyotype of a single subculturing line.

We used comparative genomic hybridization microarrays (array CGH; [43], [44]) to investigate the molecular nature of the chromosomal rearrangements. This analysis revealed that the original diploid gene copy complement was maintained for nearly the entire genome in the seven mutation accumulation lines assayed, including all four sequenced lines (∼4 KB resolution; data not shown). The only exceptions were cases of copy number variation detected at Y′ subtelomeric regions. Consequently, we used high resolution PFGE (Figure 3A) to better visualize the chromosomal rearrangements in these lines, and conducted Southern analysis using the Y′ sequence as probe (Figure 3B). This blot revealed that increases or decreases in chromosome size were always associated with a corresponding higher or lower intensity of the Y′ hybridization signal. This was clearly illustrated by chromosome I in the 5T-50 meiotic line, which is about 40 KB longer than the parental chromosome I, and showed a much stronger Y′ hybridization signal. Also consistent was the observation that the Y′ hybridization signal for chromosome IX in the parental strain was stronger compared to other chromosomes, suggesting the presence of an expanded multi-copy Y′ allele on chromosome IX. This last result suggests a mechanism for the high instability observed on this chromosome through unequal crossing over.

**Figure 3:**
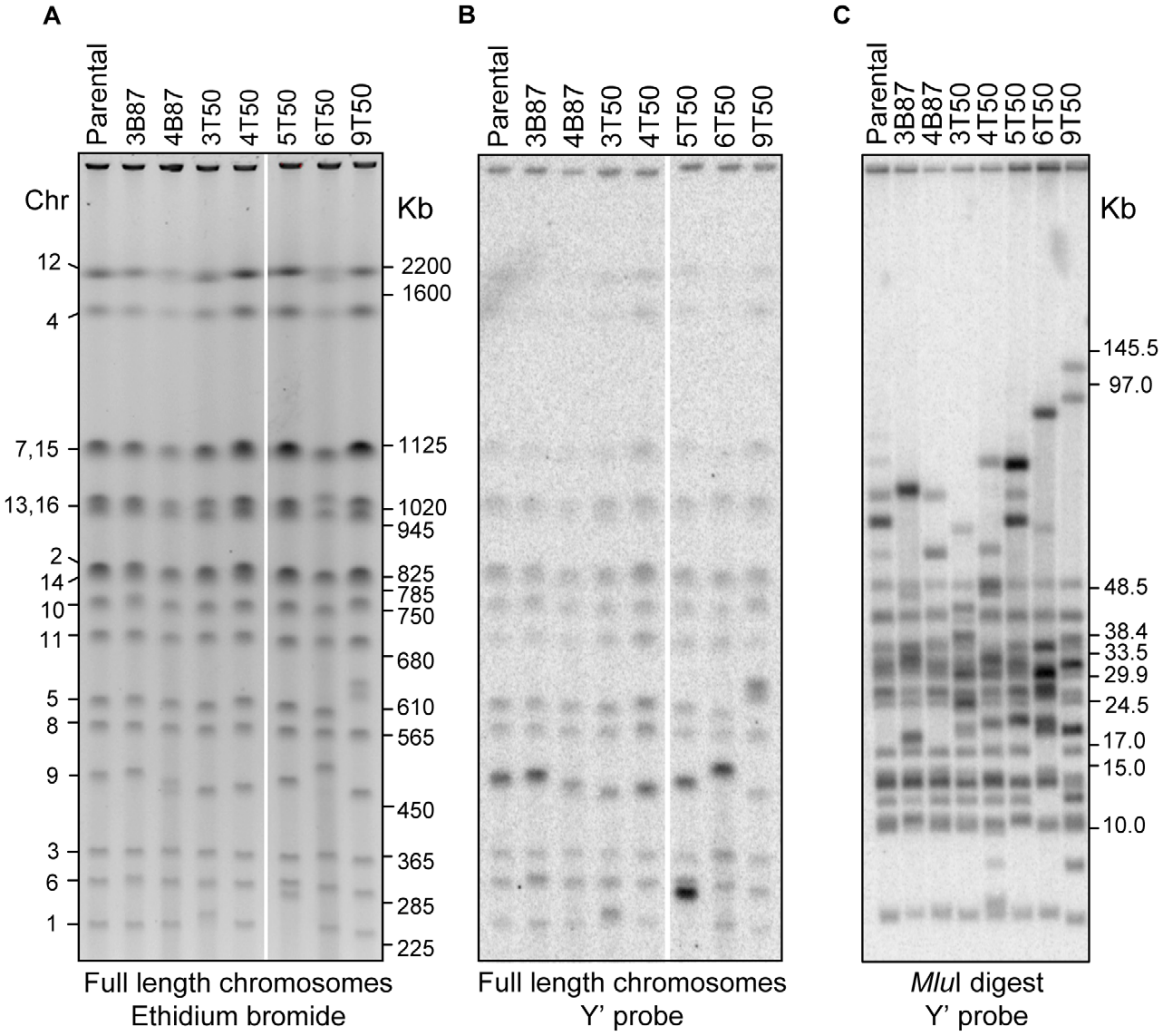
Physical analysis of chromosomes in vegetative and meiotic lines. A) High resolution PFGE of full length chromosomal DNA stained with ethidium bromide. The corresponding chromosome numbers for the parental strain are shown to the left, and the positions of BioRad *S. cerevisiae* CHEF size markers are indicated to the right (marker lane was cropped out for clarity). B) Southern blot of the PFGE in A using the Y′ sequence as probe. C) Southern blot of *Mlu*I digested genomic DNA separated in PFGE and probed with the same Y′ probe as in B. The positions of BioRad lambda CHEF size markers and NEB lambda mono-cut size markers are indicated to the right (marker lanes were cropped out for clarity).

We further investigated the involvement of Y′ sequences in the observed chromosome size variation by digesting full length chromosomal DNA with the *Mlu*I restriction endonuclease, which does not have recognition sites in Y′, and therefore releases terminal chromosomal fragments. The *Mlu*I digested DNA was separated by size with PFGE and probed with Y′ to visualize the terminal fragments (Figure 3C). This analysis uncovered additional cases of size variation that were too small in range to be resolved in chromosomal PFGE, and also narrowed down their occurrence to the regions near the ends of chromosomes. All seven strains analyzed displayed at least two chromosome ends of variant size.

Taken together, our data strongly suggest that most of the chromosomal rearrangements that accumulated in the mutation accumulation lines were due to Y′ recombination. Since the rearranged regions did not span essential genes, this result also explains why spore viability remained high in the mutation accumulation lines despite the presence of chromosomal rearrangements. While we did not investigate the specific break point structure of the Y′ rearrangements, our data suggest that none of the rearrangements involved breakpoints at internal locations. First, all chromosome size changes were associated with a corresponding increase or decrease in the hybridization signal for the Y′ probe in PFGE/Southern analysis. Second, non-reciprocal translocations associated with copy number variation were not observed in the array CGH assay. Third, the high spore viability seen for the vast majority of lines (except for those containing lethal heterozygous mutations) suggest that reciprocal translocations did not occur; such events would have likely conferred reduced spore viability. Fourth, any reciprocal translocations that formed would have to be very close in size (within 5 to 10 KB) to the parental chromosomes. Lastly, paired-end analysis would have identified such breakpoints; none were identified.

In addition to structural chromosomal aberrations, we also looked for changes in chromosome number using image tracing analysis of the PFGE profiles (data not shown). This analysis showed that for the entire data set all chromosomal bands of unchanged size were present at the same intensity relative to the parental strain (data not shown), indicating that aneuploidy never accumulated in any of the lines.

### Intra-tetrad spore-spore mating leads to rapid homozygosity of new mutations in the meiotic lines

**Figure 4:**
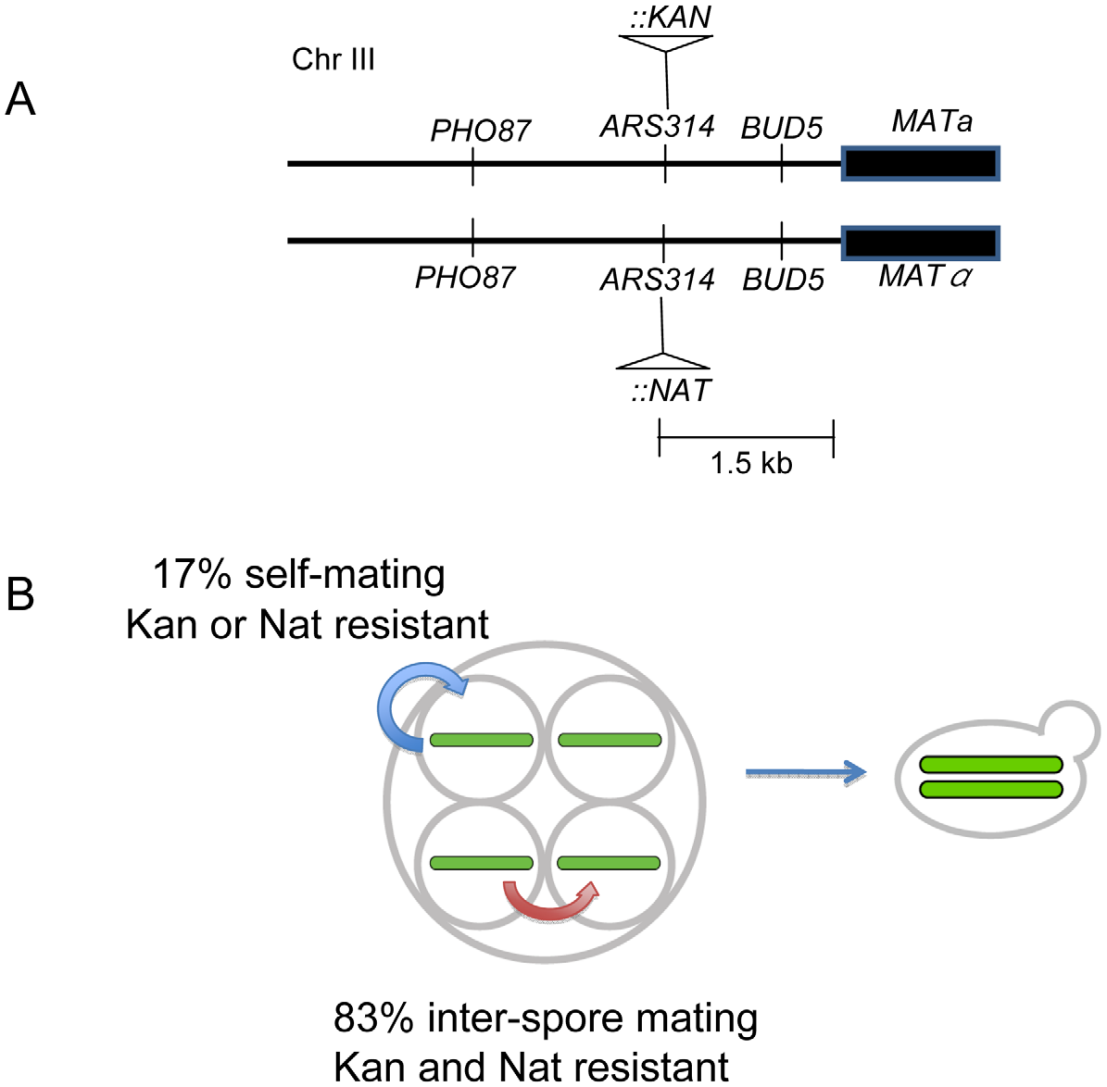
Mating patterns in *S. cerevisiae* tetrads. A) *kanMX* and *natMX* drug markers were inserted in the same site in *ARS314*, located between *PHO87* and *BUD5*, 1.5 KB proximal to *MAT*. The insertions do not disrupt either of the two genes. B) Outcomes from inter-spore and self-mating. *MATa/MATalpha* diploids that showed resistance to both antibiotics were categorized as resulting from inter-spore mating; those that showed resistance to only one antibiotic were categorized as resulting from a self-mating.

The presence of homozygous base substitutions and structural variants in the meiotic lines can be explained by the initial appearance of heterozygous mutations that are fixed to homozygous in subsequent meiotic bottlenecks by inbreeding. Self-mating through HO-induced mating-type switching [45] will immediately lead to fixation or purging of a mutation while inter-spore mating would lead to fixation or purging only in a fraction of the possible mating combinations (see below). To estimate the frequencies of self-mating and inter-spore mating, we inserted the *kanMX* and *natMX* markers at chromosome III at the *ARS314* locus that is tightly linked (1.5 KB proximal) to *MAT* in the diploid homothallic parent strain EAY2531 (Figure 4A). The introduction of these drug markers is unlikely to affect the efficiency of *MAT* locus switching because the insertions are distal to the HO-induced DSB site. Consistent with this, single spores from strains containing the *kanMX* or *natMX* insertions near *MAT* were able to switch mating type and form diploids at frequencies similar to those from strains unmarked near the *MAT* locus (data not shown). A diploid that forms by inter-spore mating will be resistant to both *G418* and nourseothricin. A diploid formed by self-mating will be resistant to *G418* or nourseothricin but not to both. Our analysis accounts for rare single crossovers (double crossovers would not affect genotyping of the diploids) that can occur between the drug markers and the *MAT* locus, yielding progeny resistant to only one drug but arising from inter-spore mating (Table S4). This was determined by creating haploid strains EAY2694 and EAY2697 in which drug markers were linked to *MAT* and the *HO* gene was disrupted. The genetic map distance between the drug markers and the *MAT* locus (1.5 KB physical distance) was 1.0 cM, suggesting that the drug marker insertions would not have a major effect on the analysis (Table S4).

Two independent diploid colonies were isolated from the single cell streak of each germinated tetrad colony of EAY2771 (relevant genotype *ARS314::kanMX/ARS314::natMX*) and tested for drug resistance to *G418* and nourseothricin. Two different methods, streaking and microdissection, were performed with similar results; we obtained an inter-spore mating frequency of 82% and self-mating frequency of 18% (Table 3). Taking into account the crossover frequency between the drug-resistant markers and the *MAT* locus (Table S4), the revised estimates for inter-spore mating and self-mating were 83% and 17% respectively (Figure 4B). Analysis of the intra-tetrad mating pattern also showed the presence of multiple types of mating within a single tetrad. For 18% of the tetrads analyzed, two single colonies arising from the same tetrad showed different patterns of drug resistance (Table 3). This indicates that the occurrence of one type of mating event does not prevent additional and different types of mating events within a single tetrad. The low frequency of self-mating indicates that it plays only a minor role in fixing mutations in our meiotic lines. The excess of homozygous mutations in the meiotic lines is likely due to random inter-spore mating during the meiotic bottlenecks. These analyses also suggested that the population size of the bottleneck in the meiotic lines is variable, between one and four.

**Table 3:** Intra-tetrad mating patterns detected in *S. cerevisiae*.

Method	Tetrads germinated	Single colonies obtained from germinated tetrads	Percent mating		
			inter-spore	self	multiple
Streak	100	200	84	16	14
Microdissection	84	145	79	21	21
Total	184	345	82	18	14–21

Single tetrads derived from EAY2771 (relevant genotype *ars314*::*kanMX MATa/ars314::natMX MATalpha*) were germinated on YPD media to form single colonies. These colonies were then restreaked to single cells on YPD media. Two independent diploid colonies were isolated either directly from the single cell streak of each germinated tetrad colony or by microdissection of unbudded cells present in a germinated tetrad colony. The two diploid colonies were phenotyped for resistance to *G418* and nourseothricin. Colonies that showed resistance to both antibiotics were categorized as inter-spore maters, while those resistant to only one antibiotic were categorized as self-maters. Multiple refers to events where one of the two diploids isolated from a single tetrad was an inter-spore mater and the other a self-mater.

In our bottleneck scheme, if the formation of a diploid cell from a germinating tetrad occurs only by inter-spore mating, a heterozygous mutation unlinked to *MAT* has a 2/3 chance to remain heterozygous in the resulting diploid, and a 1/6 chance to become mutant homozygous or wild-type homozygous (Figure S1A and S1B). After multiple rounds of meiosis followed by mating, half of the mutations that are initially heterozygous will become homozygous and half will be lost. Since we determined the proportion of inter-spore mating to be 83%, the probability of a mutation being fixed to homozygosity or lost after *n* rounds of meiosis by inter-spore mating is 1−(2/3×0.83)*^n^*. The diploids that formed by self-mating (17%) will become either fixed or lost in a single round. Based on these calculations, a new heterozygous mutation has a probability of over 99% of being fixed or lost after nine meiotic bottlenecks. The above prediction was confirmed by sequencing five of the base substitutions identified in one of the meiotic lines (3T50) at intermediate stages (10, 20, 30 and 40 rounds) of the bottleneck. All five base substitutions became homozygous mutant in 10 or fewer meiotic bottlenecks (Table S5). For two of the five mutations a heterozygous mutation could be seen in an intermediate bottleneck. Consistent with these results, in the simulation of the meiotic bottlenecks (Figure 2 and Figure S2) the observed number of heterozygous SNPs is always considerably low relative to homozygous SNPs. Our data show that heterozygous mutations will persist when propagated vegetatively but will fix very rapidly when propagated in alternating cycles of vegetative and meiotic growth due to self and spore-spore mating within a tetrad. This information will be useful to estimate how often *S. cerevisiae* undergoes a meiotic cycle based on the level of heterozygosity in wild populations (e.g. [34], [46]).

## Discussion

We used whole-genome analysis methods to compare mutational profiles of vegetative and meiotic cycles of diploid baker's yeast. We estimated the vegetative base substitution rate to be 2.9×10^−10^ per base per cell division, which is very similar to the estimate obtained by Lynch *et al.* ([17]; 3.3×10^−10^) for haploid yeast. Our analysis indicated that the meiotic mutation rate is within the range of being equal to zero to being 55-fold higher than the vegetative rate. A large number of structural variations confined to subtelomeric sequences caused by intra or interhomolog recombination events were seen in meiotic lines that did not affect their viability. Only one structural variant was observed in the five lines that were analyzed by whole-genome sequencing. In combination with the absence of in-del mutations and changes in ploidy, these results suggest a remarkable stability of the yeast diploid genome in mitosis and meiosis. We also generated a high quality SK1 yeast genome, which is likely to be a useful tool for the yeast community, given the widespread use of the SK1 strain by researchers studying meiosis. Finally, our estimates of the relative frequencies of inter-spore and self-mating will provide useful information to population geneticists for analyzing the life style of this model organism [47].

### Determination of vegetative and meiotic base substitution rates

The single base substitution rates in the meiotic lines and vegetative lines were nearly identical to each other and to rates obtained previously by Lynch *et al.* [17] working with haploid lines. Although mutagenic effects of meiosis were not observed in our meiotic lines as measured by spore viability, it is possible that at the level of a single division, meiosis is more error prone than mitosis. Because our lines alternated between 20 vegetative generations and one meiotic generation, our estimates for meiotic mutation are less precise. Based on simulations shown in Figure 2, we can assign upper limits (25 to 55-fold) on increased base mutation rates in meiosis compared to vegetative growth. A more precise genome-wide estimate would likely require a large-scale deep sequencing analysis that involves sequencing DNA from thousands of independent spore clones from a single round of meiosis. However, based on recent work in *S. paradoxus* suggesting that a meiotic cycle occurs only once per 1,000 vegetative cycles [34], it's not clear if a meiotic mutation rate at the upper limit as predicted by previous studies [10]–[12] would significantly impact fitness in baker's yeast.

During meiosis approximately 140 to 170 double-strand breaks (DSBs) are induced in a single cell [48]. These breaks are repaired through mechanisms that involve roughly 0.8 to 1.9 KB of DNA synthesis [49]. Thus as much as 500 KB of DNA is re-synthesized during DSB repair in a single cell during meiosis. Strathern *et al.* [11] have estimated that the misincorporation rate of the DNA polymerase(s) associated with mitotic DSB repair is 10^−6^ to 10^−5^; such a high rate could be responsible for the high meiotic mutation rates observed by Magni and von Borstel [10]. In the meiotic bottleneck performed in this study (50 meioses, with only 25% of the mutations recovered because repair synthesis is thought to occur on only one of the four chromosomes, and half are lost due to mating), one would expect in each line between 3 (1×10^−6^ rate) and 30 (1×10^−5^ rate) mutations associated with meiosis. Our data are compatible with a DNA polymerase misincorporation rate of 1×10^−6^, but suggest that previous upper-end estimates are too high. Alternatively, the polymerases associated with meiotic DSB repair are more accurate than those active in mitotic DSB repair, or DNA mismatch repair could more often excise DNA synthesis errors in meiotic DSB repair than in mitotic DSB repair.

### Lack of structural variants and in-dels suggests the yeast diploid genome is highly stable

No SVs larger than 500 bp (we could not detect SVs between 3 and 500 bp) were detected in single-copy coding regions or other single-copy sequences despite using methods (read depth coverage, PFGE, array CGH) that are highly sensitive to a large range of structural variant sizes. These results are in contrast to the findings of Lynch *et al.* [17] who observed that the majority of the mutational changes in the haploid vegetative lines were structural variations involving copy number changes mediated by non-allelic homologous recombination (NAHR) between dispersed repeat elements distributed throughout the genome. Another important difference is that our diploid lines did not show the genomic instabilities that were frequently observed by Lynch *et al.* [17] at different stages in their bottleneck analysis. In fact, the haploid lines in Lynch *et al.* [17] rapidly accumulated whole chromosome gains to become in effect diploids within a few hundred generations, at which point the relative chromosome number stabilized. A reverse trend was observed when yeast tetraploids submitted to a subculturing regimen progressively lost chromosomes until a stable diploid state was reached [50]. These results point to a remarkable stability of the yeast diploid genome and to the possibility that diploid genomes are inherently more resistant to chromosomal rearrangements than haploids. This occurs despite the fact that diploids are able to tolerate the deletions of large regions spanning essential genes that would otherwise be lethal in a haploid. The availability of a homologous chromosome template at all times during the cell cycle is likely to improve the fidelity of repair of chromosomal breaks, since most mitotic crossover events in diploid yeast can be explained by precursor double-strand break lesions that occur during G1, before genome replication [51]. A haploid genome would be ill equipped to repair such breaks, possibly leading to SVs similar to those observed by Lynch *et al* [17].

Small in-del mutations are thought to occur at high rates in homopolymeric runs due to replication slippage [52], [53]. As described above, we did not identify 1–2 nt in-del mutations in any of the sequenced lines. One possibility is that our methods were not sensitive enough to detect in-dels in homopolymeric tracts. Such mutations can be identified only if the entire tract and unique sequences flanking both sides are present in the short read sequence, and the alignment program can map the in-del. However, in another study our methods have been successful in identifying in-dels in five to thirteen bp homopolymeric tracts located in single copy genes (XM and CB, unpublished data). Previous estimates for frameshift mutation rates in homopolymeric tracts at the *LYS2* locus in yeast were 3.3×10^−9^, 16×10^−9^, and 47×10^−9^ for A_5_, A_8_, and A_10_ runs, respectively [52]. In a comparison of N_10_ tracts at a single locus, Gragg *et al.* [53] observed rates that varied from 24×10^−9^ for A_10_ tracts to 10,500×10^−9^ for G_10_ tracts. If we assume that all in-dels occur in homopolymeric tracts in the diploid yeast genome (154,850, 98% of which are ≤10 nt), then the rate of in-dels in our generation 87 bottleneck lines is <3.7×10^−9^, which appears lower than previous mutation rate estimates for homopolymeric tracts of five to ten nucleotides [17], [52].

While the single copy regions of the genome were highly stable, our subculturing lines showed widespread structural variation in the regions near chromosome ends with low gene content, namely in the Y′ subtelomeric repeats. Such repeats are highly variable between yeast strains and have been shown to recombine ectopically both in vegetative and meiotic cells (reviewed in [54]). Analogous dynamic structures have been well characterized in human subtelomeres as well [55]. The high rate of subtelomeric recombination mediated by both homologous and non-homologous mechanisms is thought to be responsible for the remarkable diversity of subtelomeric configurations that exist between individuals, many of which have been implicated in disease processes [56]. Taken together, our results are consistent with the high rate of structural variation at subtelomeres, and support the proposal that the peripheral regions of chromosomes are much more plastic than the gene-rich central sections of the diploid genome where structural rearrangements are associated with more severe phenotypic consequences [57], [58].

It is important to note that there are methodological differences between our study and that of Lynch *et al.* [17] in the sequencing technologies used. Their analysis involved longer sequencing reads but at lower coverage (∼5-fold, ∼50% of the genome); we obtained much deeper coverage (40 to 60-fold, ∼95% of the genome). Our sequencing approach prevented us from accurately detecting structural variations of between 3 and 500 bp. However, since the differences seen in the two studies primarily involved structural variations greater than 1 KB, the use of different technologies should not be a factor in interpreting the two data sets.

### Mating patterns in baker's yeast lead to both rapid fixation as well as purging of new mutations

Intra-tetrad mating appears to be a major component of the sexual life cycle of most yeasts, while the frequency of outcrossing in *S. cerevisiae* is estimated to be very low, once every 50,000 divisions [46], [59]. Many natural isolates of yeast are homothallic (*HO*) and are capable of switching mating type ([45]; reviewed in [60]). We showed by linkage analysis that 83% of intra-tetrad matings in homothallic *S. cerevisiae* tetrads occur by inter-spore mating. This is consistent with the high frequency of inter-spore mating (94%) that was previously inferred in *S. paradoxus* tetrads by population genetics based approaches [34]. The high frequency of spore-spore mating seen in *S. cerevisiae* may be due to the presence of inter-spore bridges that are maintained within a tetrad [61]. Self-mating might also be impeded by the requirement that the mother cell undergo two divisions before it can switch mating type [45].

Intra-tetrad mating is expected to create a drive towards homozygosity of mutations. The absence of empirical estimates for the relative frequencies of different modes of mating in a tetrad, and the experimental difficulty of tracing mutations on a genome wide scale following mating have led to considerable theory [31], [62], but little direct evidence for the relationship between mutation heterozygosity and mating pattern exists. Given the advantages for the maintenance of heterozygosity in populations [63], one might expect inter-spore mating to have a selective advantage over self-mating, since heterozygosity is lost only by a third during inter-spore mating whereas it is completely lost by self-mating [30]. By tracking mutations identified through whole genome sequencing of the meiotic mutation accumulation lines and using experimentally determined estimates of mating patterns within a tetrad, we showed that most new mutations, including base substitutions and structural variations, can go to fixation very rapidly, in less than ten rounds of meiosis and inbreeding. The ratio between mitotic and meiotic cycles in wild populations of *S. cerevisiae* is not known, although in *S. paradoxus*, population genetics approaches have determined that it undergoes a sexual cycle approximately once every 1,000 asexual cycles [34]. The information presented in this study should encourage the use of population genetic approaches to estimate how often *S. cerevisiae* undergoes a meiotic cycle (e.g. [34], [46]).

Our data provided an estimated base substitution rate of 2.9×10^−10^ (per base per cell division) for vegetative growth in diploid baker's yeast. This analysis also showed that the meiotic mutation rate in baker's yeast is within the range of being equal to zero to being 55 times higher than the vegetative rate. We observed a large number of structural variations at subtelomeric regions in vegetative and meiotic lines and did not appear to affect spore viability. Only one structural variant was observed at a non-telomeric location, and no changes in ploidy were seen. Together, these data illustrate the remarkable stability of the baker's yeast diploid genome in the vegetative and meiotic cell cycles.

## Materials and Methods

### Media

Yeast strains were grown on yeast extract-peptone-dextrose (YPD) medium [64]. When required, YPD medium was supplemented with Geneticin (*G418*, Invitrogen, San Diego) and nourseothricin (Werner BioAgents, Germany) as described previously [65], [66]. Sporulation medium was prepared as described in Argueso *et al*. [67].

### Establishment of mutation accumulation lines for mitotic and meiotic divisions

Mutation accumulation lines were initiated with the SK1 strain EAY2531 (*MATa/MATalpha*, *HO/HO*, *ura3Δ::hisG/ura3Δ::hisG*, *leu2::hisG/leu2::hisG*, *lys2/lys2*; [35]). We made this parental strain by isolating a diploid strain from a single homothallic spore derived from NKY730 (kindly provided by Nancy Kleckner, same genotype as EAY2531). Like NKY730, EAY2531 sporulates rapidly and with high efficiency and spore viability (96%). EAY2531 was streaked to single cells on solid YPD media and after 48 hrs of growth at 30°C, 20 single colonies were split into two sets of lines. One set of 20 lines was designated the vegetative bottleneck line “B” and the other, “T” was designated to undergo vegetative and meiotic cycles as explained below.

#### Vegetative “B” lines

To initiate the vegetative growth bottleneck, one half of each of the 20 starting colonies described above was streaked to single cells on solid YPD media. The single cells were grown for 48 hrs at 30°C (∼20 generations) to form a colony. One colony for each line was then restreaked and this was repeated 87 times to yield lines 1B-20B grown for ∼1740 generations. The lines were tested for their ability to grow on a non-fermentable carbon source (lactate) at intermediate stages to identify any loss of mitochondrial function because loss of mitochondrial function can cause high mutation rates [68]. No such loss was observed.

#### Meiotic “T” lines

The other half of each of the starting 20 colonies described above was streaked to single cells on solid YPD media. After growth for 48 hrs at 30°C, one colony for each line was then patched onto sporulation media and incubated for 24 hours at 30°C. A single tetrad for each line was isolated by microdissection and germinated on YPD plates (48 hrs growth at 30°C, ∼20 generations) to form a single colony. This colony was then sporulated and the bottleneck was repeated 50 times to yield lines 1T-20T grown for ∼1,000 vegetative generations and 50 meiotic generations. At the end of the bottleneck experiments, cells from the final B and T lines were sporulated and tetrad dissected to assess fitness.

### Whole genome sequencing

All strains were grown to saturation in 100 ml of YPD medium at 30°C and high quality DNA was extracted using a QIAGEN Genomic Tip according to the manufacturer's instructions. 5 µg of genomic DNA were fragmented using a Covaris DNA shearer and size-selected to ∼300 bp in a 2% agarose gel. Sequencing libraries were generated using an Illumina Genomic DNA Sample Prep Kit, according to the manufacturer's protocol. To increase coverage and allow detection of in-dels and SVs, all strains were sequenced paired-end with 36 nt reads using an Illumina Genome Analyzer GAII. Sequencing information for each strains is shown in Table S1. The sequences data were submitted to the European Read Archive (accession number ERA007227).

### Assembly of a reference SK1 genome

Short 36 nt reads of the parental EAY2531 strain, one lane of paired-end (8,101,474 pairs) and two lanes of single-end (8,295,633 reads) sequence, were used for de-novo assembly using Velvet [69] and ABySS [70] separately. First, the optimal k-mer size, for both tools, was determined by scanning the whole parameter space (kmer size from 11 nt to 31 nt) for the best assembly. The N50, the size of the longest contig, the overall number of nucleotides in the assembly, and the number of generated contigs were used as metrics to identify the best assembly. The final assembly was done with a kmer size of 27 and 29 for Velvet and ABySS, respectively. The generated contigs were then combined using the minimus2 software [71]. In total, 1,139 contigs were assembled with an N50 of 36,291 bp. These contigs were then aligned to the *SK1* genome sequence of the Saccharomyces Genome Resequencing Project (SGRP; [72]) using BLAST [73]. Gaps in the SGRP *SK1* genome were filled with the corresponding sequences from the parental EAY2531 strain, and SNP and small in-del errors were corrected. In total, 56 gaps were filled. The new SK1 haploid genome sequence has 12,241,773 bp, covering 96% of the whole S288c nuclear genome. This *SK1* sequence was used as a reference for further analyses. The reference sequence can be downloaded and also searched using BLAST at http://steinmetzlab.embl.de/SK1.

### Detection of base substitutions and small in-dels

Short reads from the parental strain and the mutation accumulation lines were separately mapped to the *SK1* references genome using the MAQ software [74]. Two mismatches were allowed for short-read alignment. For each strain, base substitutions and short in-dels were detected using the default filtering parameters of MAQ [74]. Detected polymorphisms in the mutation accumulation lines were compared to those detected in the parental strain to define strain-specific mutations. These mutations were manually checked in the alignment and finally confirmed by Sanger sequencing the 3B-87, 4B-87, 3T-50, and 4T-50 diploid lines. For the 2B-52 line, two of the five mutations were confirmed by sequencing three haploid spores derived from the 2B52 line. The three others were confirmed by sequencing the 2B52 diploid.

We also used the Novoalign (v2.05.16; http://www.novocraft.com/) software to identify in-dels in sequenced lines using the reference SK1 genome. Since none were observed, we developed a second approach to identify in-dels. We aligned the reads directly to the S288c sequenced genome (http://www.yeastgenome.org), which is 0.7% sequence divergent from the SK1 genome. We detected approximately 9000 in-dels; however most of these were seen in all of the lines, indicating that they were likely due to sequence differences between the S288c and SK1 genomes. After discarding in-dels that were detected in 9 or all 10 sequencing runs of the parental and bottleneck lines, approximately 1,000 in-dels remained. All of the short reads that covered these in-del sites were aligned back to the SK1 assembled genome. None of these in-del calls could be confirmed after alignment, indicating that they resulted from sequence differences between the S288c and SK1 genomes.

### Simulation of base substitution occurrence in the meiotic accumulation line

To estimate the upper limit of the meiotic mutation rate, the occurrence of base substitutions was simulated *in silico* taking into account the experimental setup. For each of the 20 mitoses that occurred before each meiotic bottleneck in the “T” lines, a random number of base substitutions was generated given the observed mitotic mutation rate (2.9×10^−10^) and the size of the diploid nuclear genome (24,483,546 bp). Then, base substitutions in one meiosis were generated given different putative meiotic mutation rates (*μ*). Two scenarios for the occurrence of meiotic mutations were considered: one in which mutations occur before meiotic replication and are therefore present in both strands of one of the two sister chromatids of a chromosome, and one in which mutations occur during or after meiotic replication and are present in only one of the strands of a sister chromatid of a chromosome. Once the mutations for 20 mitoses and one meiosis have been generated as described above, the meiotic bottleneck is simulated. The spores that will undergo spore-spore mating or self-mating to form the diploid cell were chosen randomly from the four chromatids considering the observed frequencies of intra-spore mating (83%) and self-mating (17%). Since one or two spores were chosen in a single meiotic bottleneck, heterozygous base substitutions can be fixed to homozygous, persist as heterozygous or be lost for the next set of mitoses and meiotic bottleneck (Figure S1). The probability of two base substitutions occurring at the same position is very low, therefore the total number of base substitutions observed at the end of the 20 mitoses and one meiosis in the spores that will form the ongoing diploid cell equals the sum of base substitutions generated in each cell division in such spores. The set of 20 mitotic divisions plus one meiosis were then repeated 50 times to simulate the 50 meiotic bottlenecks that a single line underwent.

For each of the tested meiotic mutation rates (*μ*), 10,000 of the processes consisting of 50 bottlenecks were simulated and the distribution of the resulting number of base substitutions was recorded. The *P* value of the difference between the simulated distribution of base substitutions and the observed rate in the meiotic line was estimated as the frequency of simulations with equal or lower number of base substitutions than the average of the observed values.

### Detection of structural polymorphisms

Paired end mapping was carried out using the PEMer algorithm with default parameters [39]. For the read-depth analysis, paired-end sequencing reads were aligned to the SK1 reference genome assembly using Novoalign (v2.05.16; http://www.novocraft.com/; parameters used: -rRandom -Q 0 -R 5). Only reads with an alignment quality of >125 were used for downstream analysis (>9.6 million high-quality reads for each sample). The number of aligned reads was then counted in consecutive genomic windows of predefined size. Windows of between 100 and 400 bp were tested and a final size of 200 bp (100 bp overlap) was selected since it achieved a good trade-off between resolution and noise-level. Read-depth signals were scaled using quantile normalization [75]. For each window the log2 of the ratio between the read-depth of each of the mutation accumulation lines and the read-depth of the parent was calculated. To reduce the noise level sample specific GC-correction was performed and windows with less reads than the median(read_depth) – 2×standard deviation(read_depth) were discarded. To account for the remaining waviness of the data, local regression (LOESS) was performed [76] with a span representing a region of 20 KB.

To identify consecutive windows that show abnormal log2-ratios we used two approaches, CNV-seq [40] and DEseq (http://www.bioconductor.org/packages/2.6/bioc/html/DESeq.html). CNV-seq was used with a log2-ratio threshold of ±0.48 and a *P*-value threshold of 1×10^−37^; only regions larger that 900 bp were considered. DEseq was employed without log2-ratio threshold and a *P*-value cutoff of 0.0001. Furthermore, when using DEseq, at least two abnormal windows per SV were required at most 1 KB apart from each other.

55 putative SVs in 52 different loci that ranked highest in the read-depth analysis were further analyzed by qPCR (Table S3). qPCR was performed in an ABI 7500 thermocycler using SYBR Green and standard settings (Applied Biosystems). Reactions were performed at least in triplicates and the parental and target samples were always ran in the same plate for a given primer pair. Among-sample variation in the amount of DNA used in each reaction was normalized using independent primers for the single-copy genes *BUD23* and *ERG1*. The relative copy number difference between the mutation accumulation line and the parent was calculated as the CT difference between both lines minus the CT difference in the control regions *BUD23* or *ERG1*.

### PFGE and array CGH

PFGE was conducted using a BioRad Contour-clamped homogeneous electric field (CHEF) Mapper XA system. Agarose-embedded chromosomal DNA preparation and running conditions were performed as described previously [77]. The genomic DNA used for array CGH was purified from agarose plugs prepared for PFGE, using a procedure modified from the QIAGEN QIAquick Gel Purification Kit. Briefly, four ∼70 µl agarose plugs per sample were dissolved in 840 µl of QIAGEN QG buffer. The DNA in this solution was fragmented through sonication to a size of 1–2 KB, and 280 µl of isopropanol were added. The mixture was bound to QIAquick columns, washed with QIAGEN PE buffer, and eluted in 32 µl of QIAGEN EB buffer. This procedure yielded 2–3 µg of fragmented DNA which was labeled and hybridized for array CGH assays as described previously [78].

### Determination of intra-tetrad mating pattern

EAY2771 (*HO/HO*, *ars314::kanMX MATa/ars314::natMX MATalpha*, *ura3Δ::hisG/ura3Δ::hisG*, *leu2::hisG/leu2::hisG*, *lys2/lys2*) was constructed by sequentially inserting *kanMX* and *natMX* drug resistance markers into identical positions at *ARS314* in EAY2531. This locus is 1.5 KB proximal to the *MAT* locus. 184 tetrads obtained by sporulating EAY2771 were placed by microdissection at unique positions on a YPD plate. All 184 tetrads germinated and formed colonies on YPD. The intra-tetrad mating pattern was determined using two approaches. In the first approach, 100 of the colonies were re-streaked on YPD plates to single cells. Two of the resulting colonies were patched onto YPD-*G418* and YPD-nourseothricin to assess antibiotic resistance, and onto sporulation plates to assess ploidy (all were diploids). In the second approach, the remaining 84 colonies were streaked onto YPD to single cells and two unbudded cells from each original colony were isolated under the dissection microscope. These single cells were incubated on YPD to form colonies. 145 of these cells formed colonies. The resulting colonies were patched onto YPD plates containing *G418* or nourseothricin to assess antibiotic resistance, and onto sporulation plates to assess ploidy (all were diploids). The second approach was performed to eliminate the possibility of closely spaced multiple cells from the restreak giving rise to single isolated colonies.

### Estimating crossover frequency between the drug-resistant markers and the *MAT* locus

EAY2775 (*ars314::kanMX MATa/ars314::natMX MATalpha*, *ho::hisG-URA3-hisG/HO*, *ura3Δ::hisG/ura3Δ::hisG*, *leu2::hisG/leu2::hisG*, *lys2/lys2*) is a derivative of EAY2771 in which the *HO* gene was disrupted with the *hisG-URA3-hisG* marker. Haploid segregants of EAY2775, EAY2694 (*ars314::kanMX MATa*, *ho::hisG-URA3-hisG*) and EAY2697 (*ars314::natMX MATalpha*, *ho::hisG-URA3-hisG*), were mated on complete plates for four hours and then transferred to sporulation medium for 48 hrs. Tetrads were dissected on YPD medium and incubated at 30°C for 48 hrs. Spore clones were replica plated onto selective media and mating testers and segregation data from each replica was analyzed using the RANA software [67]. The *kanMX* and *natMX* markers (EAY2771) each showed 2∶2 segregation and segregated independently from each other in all tetrads analyzed. No gene conversion events involving the drug resistance markers were seen.

## Supporting Information

Figure S1 Schematic description of the different scenarios of SNP occurrence and their fixation in the meiotic bottlenecks considered for the simulations. Mutations are depicted as red stars and meiosis is depicted by an ascus as in Figure 1. The probability that a mutation is fixed (*P_F_*), lost (*P_L_*), or remains as heterozygous (*P_H_*) is given in the equations at the bottom of the figure. In the equations, *s* represents the proportion of spores that undergo self-mating. A) Heterozygous mutation present before meiosis, occurred during preceding mitoses or meioses. B) Mutation occurred in meiosis before meiotic replication. C) Mutation occurred in meiosis during or after DNA replication.
(0.20 MB TIF)

Figure S2 Detailed results of the simulation to estimate the upper limit for the meiotic mutation rate. As in Figure 2 of the main text, the histograms show the distribution of the final number of homozygous (white) and heterozygous (grey) mutations that occurred in 10,000 independent simulated lines after 1,000 mitotic divisions and 50 meiotic bottlenecks in each line. The putative meiotic and mitotic mutation rate used for each of the simulation is shown on top of each histogram. The red vertical lines show the average number of SNPs (all homozygous) observed in the T50 lines. The *P*-value denotes the frequency of simulations with equal or lower number of SNPs than the observed value. Panel A shows simulations in which meiotic mutations were set to occur only before DNA replication and therefore are present in two chromatids (as in Figure 2A and 2B). Panel B shows simulations in which meiotic mutations were set to occur during or after DNA replication and are therefore present in one single chromatid (as in Figure 2C and 2D). See Material and Methods and Figure S1 for further details on the simulations. In the histograms, since heterozygous SNPs are rapidly fixed to homozygous or lost in the meiotic bottlenecks, the frequency of heterozygous SNPs is always relatively low.
(0.67 MB TIF)

Figure S3 PCR mapping of the Ty3 element insertion in the ribosomal DNA of 3T50. A) Schematic map of the ribosomal DNA repeat unit (9.1 KB) and of the Ty3 retrotransposable element with its two flanking s LTRs (5.4 KB). The rDNA is present in chromosome XII as a tandem array of 100 to 150 repeat units; one complete unit is shown. Primers specific for the rDNA and Ty3 regions are shown as black arrows, the direction of the arrowheads correspond to their 5′ to 3′ orientation. B) Primers specific to the Ty3 sequence were used in conjunction with primers in the ribosomal DNA repeat unit. Non-specific PCR products are seen in the SK1 parental strain, in the 3T50 line, and the independent subculturing line 3B87. These non-specific products are likely present due to the highly repetitive nature of the rDNA region. PCR products specific to the 3T50 line (*) were obtained with the Ty3 reverse primer JAO474 (5′TCGAGGTAGTCTTGCGCCAGG3′) and reverse rDNA primers, indicating that the Ty3 element is inserted in Crick orientation relative to the rest of chromosome XII. The smallest product was obtained with rDNA primer 8R (5′AGCGGCAAACATGAGTGCTT3′), therefore the insertion is present near the 5′ end of the rDNA repeat unit. C) The site of insertion was further narrowed further by using the forward Ty3 primer JAO473 (5′ACGTAAGGCGAGTTCTAACCG3′) and the rDNA9F (5′ CTGTCATATCCTATTGCTATTAG3′) forward primer to obtain a ∼4.3 KB PCR product (**). D) The sequences of the PCR products containing the rDNA-Ty3 left and right junctions were determined by Sanger sequencing and the respective chromatograms are shown. The new Ty3 element in 3T50 inserted at chromosome XII coordinate 459675 of the *S. cerevisiae* reference genome, one base pair upstream of the transcription start site of the RDN5 gene that encodes the 5S ribosomal subunit. This insertion also resulted in the duplication of a 5 bp sequence (ACTAT - shaded in light blue) immediately upstream of *RDN5*.
(1.17 MB TIF)

Figure S4 PFGE of full length chromosomal DNA stained with ethidium bromide for the parental diploid strain and for all twenty vegetative mutation accumulation lines. The corresponding chromosome numbers for the parental strain are shown to the left. The chromosome size changes in each mutation accumulation line are indicated above their corresponding PFGE lane.
(1.17 MB TIF)

Figure S5 PFGE of full length chromosomal DNA stained with ethidium bromide for the parental diploid strain and for all nineteen meiotic mutation accumulation lines. The corresponding chromosome numbers for the parental strain are shown to the left. The chromosome size changes in each mutation accumulation line are indicated above their corresponding PFGE lane.
(1.12 MB TIF)

Table S1 Illumina Genome Analyzer data. All strains (diploid or spore clone derivatives) were sequenced with 36 nt reads. ^a^Percentage of the SK1 genome covered by at least three reads.
(0.04 MB DOC)

Table S2 Spore viability of vegetative and meiotic mutation accumulation lines. Spore viability for the 1B-87 to 20B-87 and 1T-50 to 20T-50 lines was determined by sporulating the final bottleneck strain for each line and then tetrad dissecting 20 tetrads per line on rich media. For lines 3B-87, 4B-87, 3T-50 and 4T-50 spore viability was determined by dissecting 100 tetrads.
(0.04 MB DOC)

Table S3 Primers used for qPCR verification of putative SVs. ^a^ The Start and End coordinates refer to the region where the putative SV was detected. The primers bind within this region.
(0.39 MB DOC)

Table S4 Crossover frequency between markers inserted at *ARS314* and the *MAT* locus. EAY2694 (relevant genotype *ars314::kanMX MATa*, *ho::URA3*) was mated with EAY2697 (relevant genotype *ars314::natMX MATalpha ho::URA3*). The resulting diploid was sporulated and 317 tetrads were dissected. Spores were genotyped for mating type and antibiotic resistance. Recombination frequencies (Rf) in single spores were calculated as recombinant/(parental+recombinant). Genetic distance in the tetrad (cM) was calculated using the formula of Perkins [1]; 50×{TT+(6×NPD)}/(PD+TT+NPD). No gene conversion events involving the *kanMX/natMX* drug markers were seen. 1. Perkins DD (1949) Biochemical mutants in the smut fungus *Ustilago maydis*. Genetics 34: 607–626.
(0.04 MB DOC)

Table S5 Inter-spore mating within tetrads leads to rapid homozygosity of mutations in the meiotic 3T-50 line. Earlier generations of the 3T line (3T-10, 3T-20, 3T-30, 3T-40) were examined by Sanger sequencing for the presence of the five homozygous base substitutions seen in the 3T-50 line (Table 1; Materials and Methods). The DNA sequence detected in an earlier line is displayed as either parental or derived.
(0.04 MB DOC)
