# Supplementary figures and images for: The Baker's Yeast Diploid Genome Is Remarkably Stable in Vegetative Growth and Meiosis
Source: PLoS Genet. 2010 Sep 9;6(9):e1001109. doi: 10.1371/journal.pgen.1001109 (PMC2936533; doi:10.1371/journal.pgen.1001109)

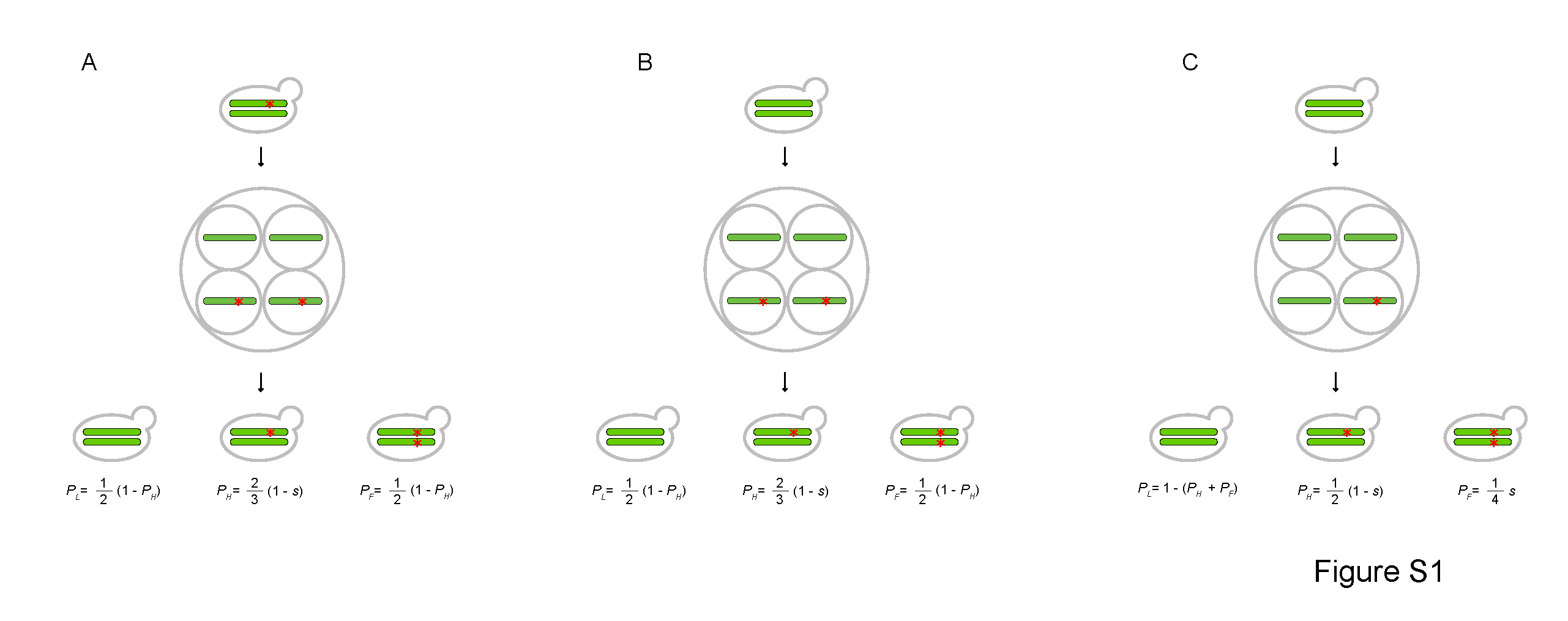

Supplement: Figure S1 — Schematic description of the different scenarios of SNP occurrence and their fixation in the meiotic bottlenecks considered for the simulations. Mutations are depicted as red stars and meiosis is depicted by an ascus as in Figure 1. The probability that a mutation is fixed (PF), lost (PL), or remains as heterozygous (PH) is given in the equations at the bottom of the figure. In the equations, s represents the proportion of spores that undergo self-mating. A) Heterozygous mutation present before meiosis, occurred during preceding mitoses or meioses. B) Mutation occurred in meiosis before meiotic replication. C) Mutation occurred in meiosis during or after DNA replication. (0.20 MB TIF) [file pgen.1001109.s001.tif]

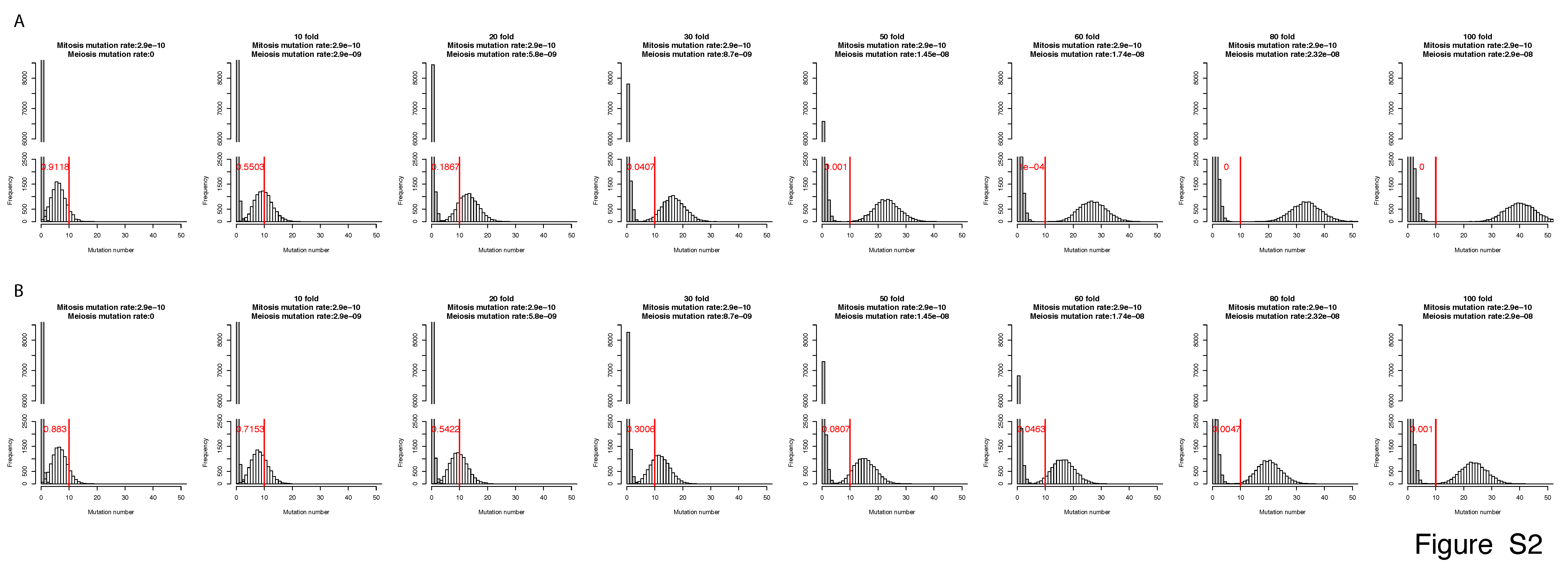

Supplement: Figure S2 — Detailed results of the simulation to estimate the upper limit for the meiotic mutation rate. As in Figure 2 of the main text, the histograms show the distribution of the final number of homozygous (white) and heterozygous (grey) mutations that occurred in 10,000 independent simulated lines after 1,000 mitotic divisions and 50 meiotic bottlenecks in each line. The putative meiotic and mitotic mutation rate used for each of the simulation is shown on top of each histogram. The red vertical lines show the average number of SNPs (all homozygous) observed in the T50 lines. The P-value denotes the frequency of simulations with equal or lower number of SNPs than the observed value. Panel A shows simulations in which meiotic mutations were set to occur only before DNA replication and therefore are present in two chromatids (as in Figure 2A and 2B). Panel B shows simulations in which meiotic mutations were set to occur during or after DNA replication and are therefore present in one single chromatid (as in Figure 2C and 2D). See Material and Methods and Figure S1 for further details on the simulations. In the histograms, since heterozygous SNPs are rapidly fixed to homozygous or lost in the meiotic bottlenecks, the frequency of heterozygous SNPs is always relatively low. (0.67 MB TIF) [file pgen.1001109.s002.tif]

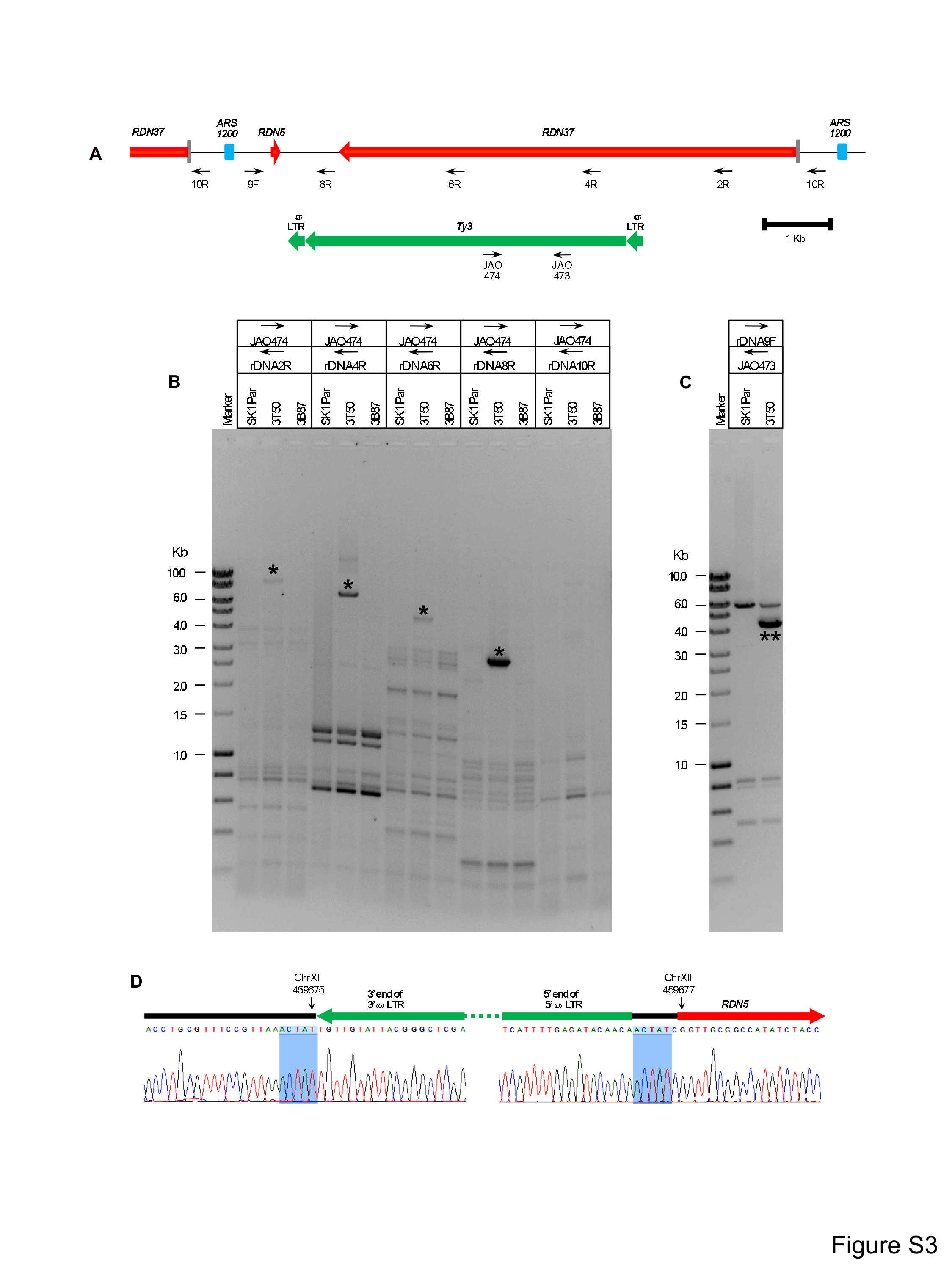

Supplement: Figure S3 — PCR mapping of the Ty3 element insertion in the ribosomal DNA of 3T50. A) Schematic map of the ribosomal DNA repeat unit (9.1 KB) and of the Ty3 retrotransposable element with its two flanking s LTRs (5.4 KB). The rDNA is present in chromosome XII as a tandem array of 100 to 150 repeat units; one complete unit is shown. Primers specific for the rDNA and Ty3 regions are shown as black arrows, the direction of the arrowheads correspond to their 5′ to 3′ orientation. B) Primers specific to the Ty3 sequence were used in conjunction with primers in the ribosomal DNA repeat unit. Non-specific PCR products are seen in the SK1 parental strain, in the 3T50 line, and the independent subculturing line 3B87. These non-specific products are likely present due to the highly repetitive nature of the rDNA region. PCR products specific to the 3T50 line (*) were obtained with the Ty3 reverse primer JAO474 (5′TCGAGGTAGTCTTGCGCCAGG3′) and reverse rDNA primers, indicating that the Ty3 element is inserted in Crick orientation relative to the rest of chromosome XII. The smallest product was obtained with rDNA primer 8R (5′AGCGGCAAACATGAGTGCTT3′), therefore the insertion is present near the 5′ end of the rDNA repeat unit. C) The site of insertion was further narrowed further by using the forward Ty3 primer JAO473 (5′ACGTAAGGCGAGTTCTAACCG3′) and the rDNA9F (5′ CTGTCATATCCTATTGCTATTAG3′) forward primer to obtain a ∼4.3 KB PCR product (**). D) The sequences of the PCR products containing the rDNA-Ty3 left and right junctions were determined by Sanger sequencing and the respective chromatograms are shown. The new Ty3 element in 3T50 inserted at chromosome XII coordinate 459675 of the S. cerevisiae reference genome, one base pair upstream of the transcription start site of the RDN5 gene that encodes the 5S ribosomal subunit. This insertion also resulted in the duplication of a 5 bp sequence (ACTAT - shaded in light blue) immediately upstream of RDN5. (1.17 MB TIF) [file pgen.1001109.s003.tif]

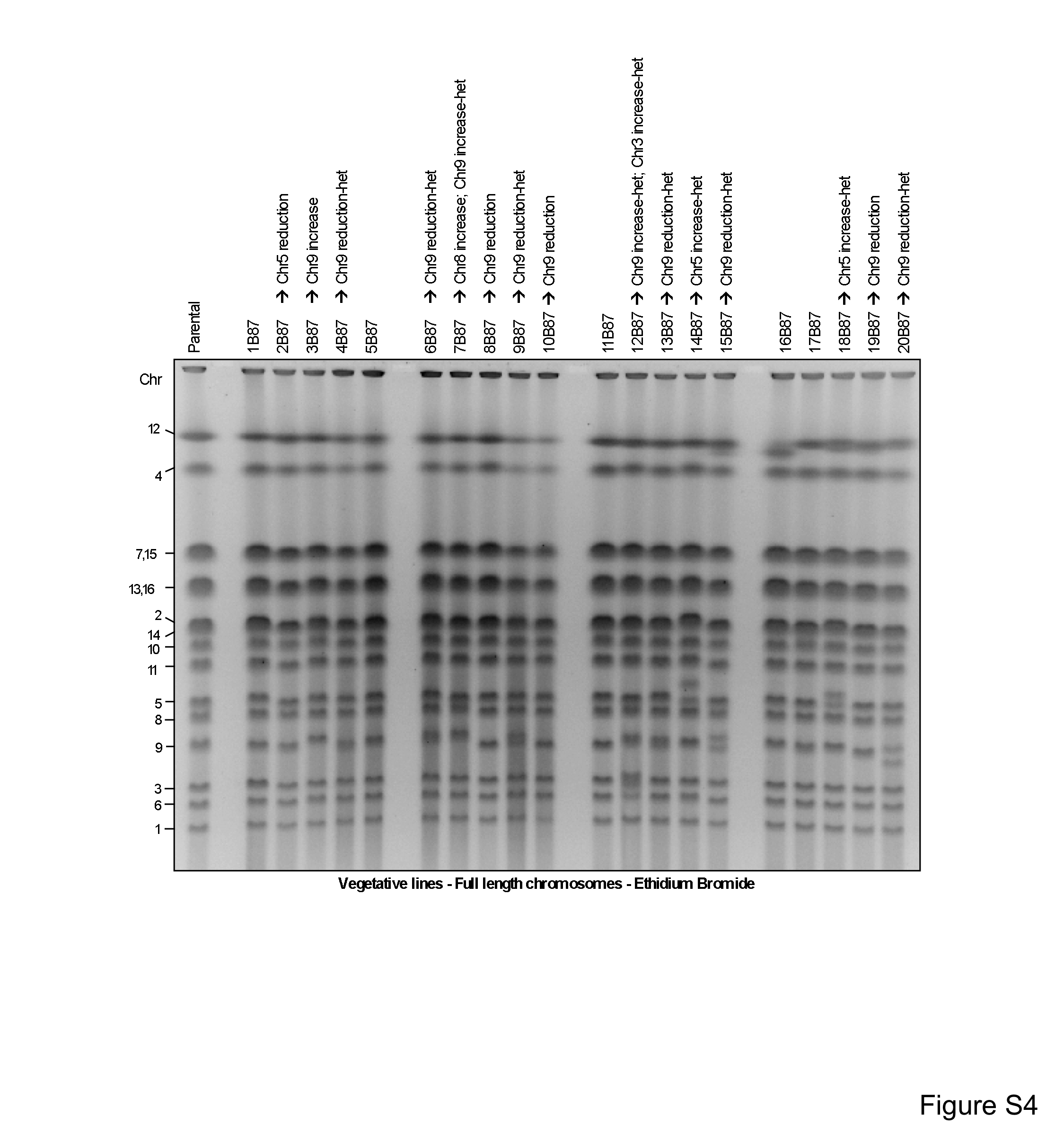

Supplement: Figure S4 — PFGE of full length chromosomal DNA stained with ethidium bromide for the parental diploid strain and for all twenty vegetative mutation accumulation lines. The corresponding chromosome numbers for the parental strain are shown to the left. The chromosome size changes in each mutation accumulation line are indicated above their corresponding PFGE lane. (1.17 MB TIF) [file pgen.1001109.s004.tif]

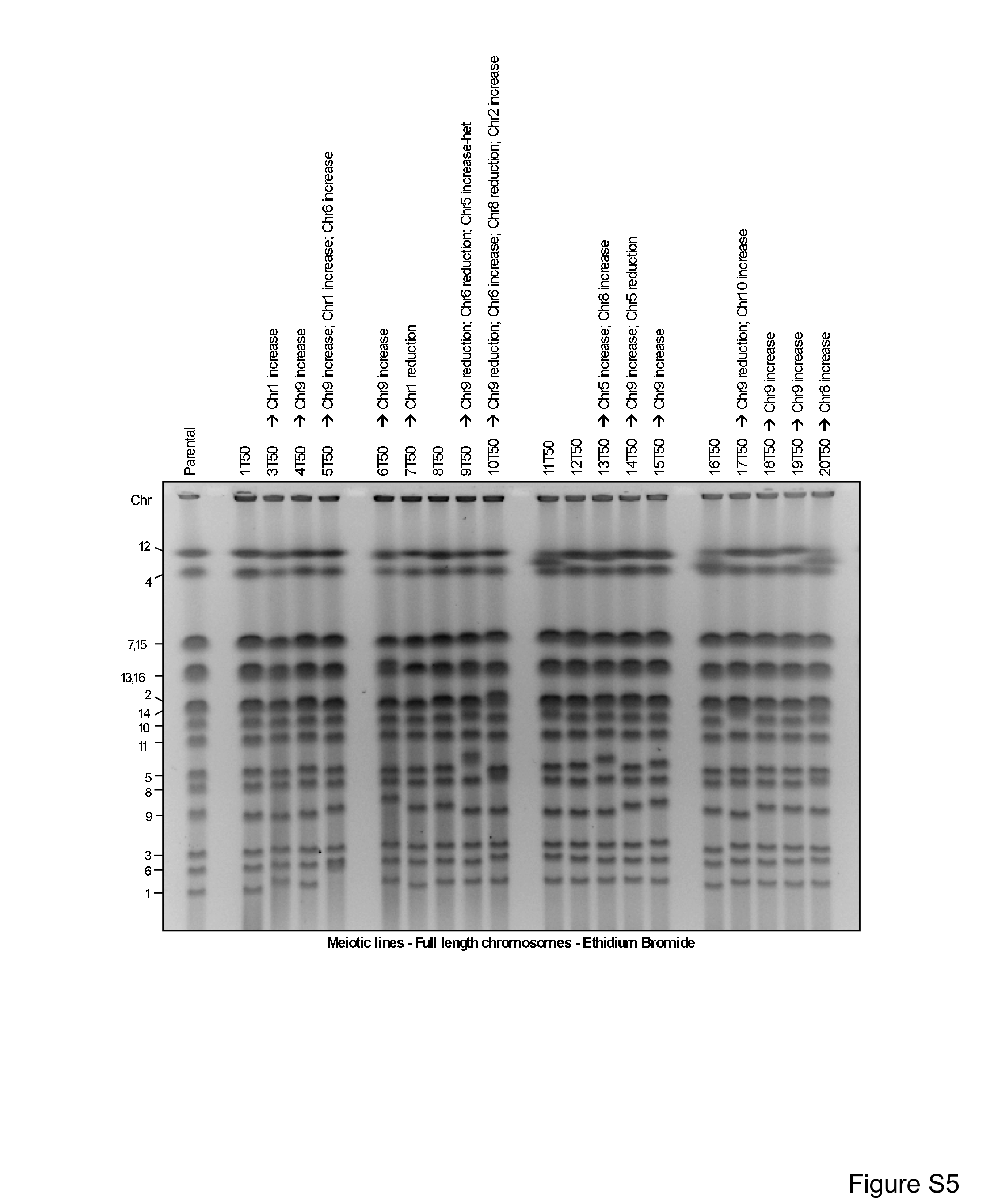

Supplement: Figure S5 — PFGE of full length chromosomal DNA stained with ethidium bromide for the parental diploid strain and for all nineteen meiotic mutation accumulation lines. The corresponding chromosome numbers for the parental strain are shown to the left. The chromosome size changes in each mutation accumulation line are indicated above their corresponding PFGE lane. (1.12 MB TIF) [file pgen.1001109.s005.tif]
