# Supplementary material for: The Baker's Yeast Diploid Genome Is Remarkably Stable in Vegetative Growth and Meiosis
Source: PLoS Genet. 2010 Sep 9;6(9):e1001109. doi: 10.1371/journal.pgen.1001109 (PMC2936533; doi:10.1371/journal.pgen.1001109)
Supplement: Table S1 — Illumina Genome Analyzer data. All strains (diploid or spore clone derivatives) were sequenced with 36 nt reads. aPercentage of the SK1 genome covered by at least three reads. (0.04 MB DOC) [file pgen.1001109.s006.doc]

**Table S1. Illumina Genome Analyzer data.**

Line Single/paired end # of reads Coverage ^a^Percentage Ploidy

______________________________________________________________________________

Parental paired 8,101,474 x 2 63.76 95.73 diploid

Parental single 8,295,633

2B-52 single 8,276,302 20.75 95.44 diploid

3B87-1 paired 8,799,486 x 2 46.16 95.66 haploid

4B87-1 paired 7,153,064 x 2 40.42 95.71 haploid

3T50-1 paired 9,014,200 x 2 49.38 95.69 haploid

3T50-2 paired 9,059,700 x 2 49.57 95.71 haploid

3T50-3 paired 8,295,590 x 2 47.21 95.71 haploid

4T50-1 paired 7,299,053 x 2 41.47 95.70 haploid

All strains (diploid or spore clone derivatives) were sequenced with 36 nt reads. ^a^Percentage of the SK1 genome covered by at least three reads.
