# Supplementary material for: The Baker's Yeast Diploid Genome Is Remarkably Stable in Vegetative Growth and Meiosis
Source: PLoS Genet. 2010 Sep 9;6(9):e1001109. doi: 10.1371/journal.pgen.1001109 (PMC2936533; doi:10.1371/journal.pgen.1001109)
Supplement: Table S2 — Spore viability of vegetative and meiotic mutation accumulation lines. Spore viability for the 1B-87 to 20B-87 and 1T-50 to 20T-50 lines was determined by sporulating the final bottleneck strain for each line and then tetrad dissecting 20 tetrads per line on rich media. For lines 3B-87, 4B-87, 3T-50 and 4T-50 spore viability was determined by dissecting 100 tetrads. (0.04 MB DOC) [file pgen.1001109.s007.doc]

**Table S2. Spore viability of vegetative and meiotic mutation accumulation lines.**

**Line % Spore Viability**

**________________________________________**

**Parental Strain**

EAY2531 96

**Vegetative Lines**

1B-87 39

2B-87 95

3B-87 97

4B-87 95

5B-87 90

6B-87 99

7B-87 100

8B-87 49

9B-87 100

10B-87 34

11B-87 97

12B-87 97

13B-87 95

14B-87 97

15B-87 100

16B-87 95

17B-87 100

18B-87 100

19B-87 94

20B-87 95

**Meiotic Lines**

1T-50 100

2T-50 Line discontinued due to contamination

3T-50 94

4T-50 98

5T-50 100

6T-50 99

7T-50 96

8T-50 100

9T-50 95

10T-50 100

11T-50 99

12T-50 97

13T-50 95

14T-50 99

15T-50 95

16T-50 99

17T-50 92

18T-50 99

19T-50 97

20T-50 99

Spore viability for the 1B-87 to 20B-87 and 1T-50 to 20T-50 lines was determined by sporulating the final bottleneck strain for each line and then tetrad dissecting 20 tetrads per line on rich media. For lines 3B-87, 4B-87, 3T-50 and 4T-50 spore viability was determined by dissecting 100 tetrads.
