# Supplementary material for: The Baker's Yeast Diploid Genome Is Remarkably Stable in Vegetative Growth and Meiosis
Source: PLoS Genet. 2010 Sep 9;6(9):e1001109. doi: 10.1371/journal.pgen.1001109 (PMC2936533; doi:10.1371/journal.pgen.1001109)
Supplement: Table S3 — Primers used for qPCR verification of putative SVs. a The Start and End coordinates refer to the region where the putative SV was detected. The primers bind within this region. (0.39 MB DOC) [file pgen.1001109.s008.doc]

**Table S3. Primers used for qPCR verification of putative SVs.**

**
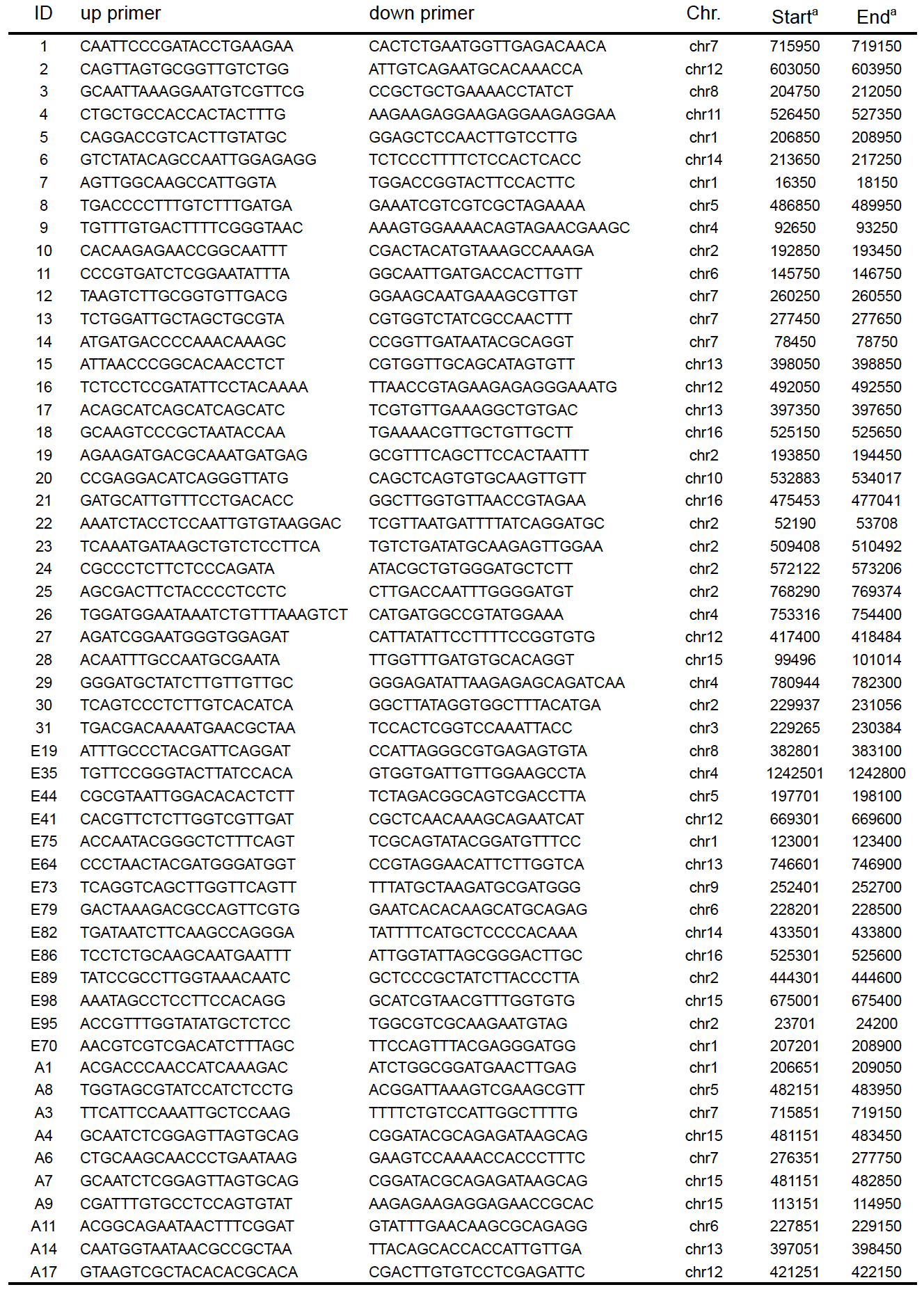
**

^a^ The Start and End coordinates refer to the region where the putative SV was detected. The primers bind within this region.
