# Supplementary material for: The Baker's Yeast Diploid Genome Is Remarkably Stable in Vegetative Growth and Meiosis
Source: PLoS Genet. 2010 Sep 9;6(9):e1001109. doi: 10.1371/journal.pgen.1001109 (PMC2936533; doi:10.1371/journal.pgen.1001109)
Supplement: Table S4 — Crossover frequency between markers inserted at ARS314 and the MAT locus. EAY2694 (relevant genotype ars314::kanMX MATa, ho::URA3) was mated with EAY2697 (relevant genotype ars314::natMX MATalpha ho::URA3). The resulting diploid was sporulated and 317 tetrads were dissected. Spores were genotyped for mating type and antibiotic resistance. Recombination frequencies (Rf) in single spores were calculated as recombinant/(parental+recombinant). Genetic distance in the tetrad (cM) was calculated using the formula of Perkins [1]; 50×{TT+(6×NPD)}/(PD+TT+NPD). No gene conversion events involving the kanMX/natMX drug markers were seen. 1. Perkins DD (1949) Biochemical mutants in the smut fungus Ustilago maydis. Genetics 34: 607–626. (0.04 MB DOC) [file pgen.1001109.s009.doc]

**Table S4. Crossover frequency between markers inserted at *ARS314* and the *MAT* locus.**

Single Spores Tetrads

Strain Parental Recombinant Rf PD TT NPD cM

____________________________________________________________________________

EAY2694/ 1213 12 0.01 289 6 0 1.0

EAY2697 ____________________________________________________________________________

EAY2694 (relevant genotype *ars314::kanMX MATa, ho::URA3*) was mated with EAY2697 (relevant genotype *ars314::natMX MATalpha ho::URA3*). The resulting diploid was sporulated and 317 tetrads were dissected. Spores were genotyped for mating type and antibiotic resistance. Recombination frequencies (Rf) in single spores were calculated as recombinant/ (parental+recombinant). Genetic distance in the tetrad (cM) was calculated using the formula of Perkins [1]; 50 x {TT + (6 x NPD)} / (PD + TT + NPD). No gene conversion events involving the *kanMX* /*natMX* drug markers were seen.

1. Perkins DD (1949) Biochemical mutants in the smut fungus *Ustilago maydis*. Genetics 34: 607-626.
