# Supplementary material for: The Baker's Yeast Diploid Genome Is Remarkably Stable in Vegetative Growth and Meiosis
Source: PLoS Genet. 2010 Sep 9;6(9):e1001109. doi: 10.1371/journal.pgen.1001109 (PMC2936533; doi:10.1371/journal.pgen.1001109)
Supplement: Table S5 — Inter-spore mating within tetrads leads to rapid homozygosity of mutations in the meiotic 3T-50 line. Earlier generations of the 3T line (3T-10, 3T-20, 3T-30, 3T-40) were examined by Sanger sequencing for the presence of the five homozygous base substitutions seen in the 3T-50 line (Table 1; Materials and Methods). The DNA sequence detected in an earlier line is displayed as either parental or derived. (0.04 MB DOC) [file pgen.1001109.s010.doc]

**Table S5. Inter-spore mating within tetrads leads to rapid homozygosity of mutations in the meiotic 3T-50 line.**

**Mutation Meiotic Line Parental sequence Derived mutation**

**__________________________________________________________**

1 3T-10 A/A

3T-20 A/A

3T-30 A/A

3T-40 A/A

3T-50 G/G

2 3T-10 G/G

3T-20 G/G

3T-30 G/G

3T-40 G/G

3T-50 T/T

3 3T-10 A/A

3T-20 A/A

3T-30 A/A

3T-40 A/G

3T-50 G/G

4 3T-10 G/G

3T-20 A/A

3T-30 A/A

3T-40 A/A

3T-50 A/A

5 3T-10 A/A

3T-20 A/A

3T-30 A/T

3T-40 T/T

3T-50 T/T

_____________________________________________________________________________

Earlier generations of the 3T line (3T-10, 3T-20, 3T-30, 3T-40) were examined by Sanger sequencing for the presence of the five homozygous base substitutions seen in the 3T-50 line (Table 1; Materials and Methods). The DNA sequence detected in an earlier line is displayed as either parental or derived.
